# Annexin A2 facilitates Japanese encephalitis virus replication by regulating endocytosis and intracellular transport in PK15 cells

**DOI:** 10.3389/fmicb.2026.1858383

**Published:** 2026-08-07

**Authors:** Longqin Wei, Saixiao Li, Chunyu Wang, Hong Zhao, Wei Luo, Jinsong Li, Lanzhen Huang, Xishi Huang, Lantao Gu, Qingli Fang

**Affiliations:** 1 School of Laboratory Medicine and Biotechnology, Guilin Medical University, Guilin, Guangxi, China; 2 Research Service Department, Guilin People's Hospital, Guilin, Guangxi, China; 3 Department of Pharmacology, Guilin Medical University, Guilin, China; 4 Scientific Research Center, Guilin Medical University, Guilin, Guangxi, China; 5 Guilin Animal Disease Prevention and Control Center, Guilin, Guangxi, China; 6 Guangxi Key Laboratory of Multimodal Biomarkers and Precision Diagnosis, Guilin Medical University, Guilin, Guangxi, China

**Keywords:** annexin A2, clathrin-mediated endocytosis, CRISPR/Cas9, intracellular transport, Japanese encephalitis virus, PK15 cells

## Abstract

**Introduction:**

Japanese encephalitis virus (JEV) is a mosquito-borne flavivirus that causes severe encephalitis in humans and reproductive disorders in pigs. However, the host factors that regulate JEV infection and replication remain incompletely understood.

**Method:**

In this study, we used porcine kidney cells (PK15) infected with the attenuated JEV strain SA14-14-2 to identify host proteins interacting with the JEV envelope (E) protein by immunoprecipitation coupled with LC-MS/MS.

**Results:**

Annexin A2 (ANXA2) was identified as a candidate host factor associated with JEV infection. ANXA2 expression was increased during JEV infection, and co- immunoprecipitation confirmed its association with the JEV E protein. Inhibition of ANXA2 with antibody reduced JEV RNA replication in a dose-dependent manner. To further define its function, ANXA2 knockout (KO) PK15 cells were generated using CRISPR/Cas9. Compared with wild-type cells, ANXA2-KO cells showed significantly reduced viral RNA accumulation, especially during the early stage of JEV infection (2-6hpi), when the viral entry and initiates replication, lower JEV E protein expression, and decreased progeny virus titers. In addition, ANXA2 deficiency altered the expression of clathrin-mediated endocytosis- related genes and proteins, including CLTC, CLTB, AP2M1, and EPS15, and affected Akt phosphorylation during early infection. Fluorescence recovery after photobleaching analysis (FRAP) of DiD-labeled JEV particles indicated impaired intracellular transport in ANXA2-KO cells.

**Discussion:**

these findings identify ANXA2 as a host factor that promotes JEV infection and replication, at least in part by regulating clathrin-mediated endocytosis and intracellular transport. ANXA2 may represent a potential target for future antiviral strategies against JEV.

## Highlights

- Annexin A2 (ANXA2) was identified as a host factor that promotes JEV infection and replication.
- ANXA2 Knockout significantly affected the infection and replication of JEV in PK15 cells, and cause the dysregulation of host clathrin-mediated endocytosis-related proteins expression, and also affected Akt phosphorylation during early infection of JEV.
- ANXA2 Knockout impaired the intracellular transport of JEV particles in PK15 cells, indicating that ANXA2 may facilitate the intracellular trafficking of viral particle by mediating the host clathrin-dependent endocytosis pathway, to promote JEV infection and replication processes

## 1 Introduction

Japanese encephalitis virus (JEV) is a mosquito-borne flavivirus belonging to the family Flaviviridae and genus Flavivirus. According to the World Health Organization, JEV remains a leading cause of viral encephalitis in humans in several Asian countries, causing an estimated 69,000 severe cases and 13,600–20,400 deaths annually despite widespread vaccination programs (Zhao et al., 2020). Although substantial progress has been made in prevention and control, JEV continues to pose a major public health burden in northern Oceania and in South, East, and Southeast Asia. After transmission by mosquito bite, JEV initially replicates at the inoculation site and then spreads to regional lymph nodes and surrounding tissues. In subclinical infections, viremia is usually limited or cleared before the virus invades the central nervous system (CNS). In clinical cases, however, the virus crosses the blood-brain barrier and causes encephalitis. In addition to its importance in human disease, JEV is a major pathogen associated with encephalitis and reproductive disorders in pigs. Domestic pigs have been identified as potential amplification hosts following JEV introduction (Park et al., 2018). Infected pigs typically develop high levels of viremia and are often asymptomatic as adults; however, abortion, stillbirth, and congenital abnormalities, including central nervous system malformations, are frequently observed in pregnant sows (Zheng et al., 2019). Therefore, JEV is not only a threat to public health and biosecurity but also causes considerable economic losses to the pork industry, particularly in areas with low vaccine coverage or limited diagnostic capacity (Shi et al., 2019).

JEV is a small, icosahedral, enveloped virus with a diameter of approximately 50 nm. Its positive-sense single-stranded RNA genome is approximately 11 kb in length and contains a single open reading frame (ORF) that is translated into a polyprotein. This polyprotein is processed into three structural proteins, capsid (C), membrane (M), and envelope (E), as well as seven non-structural proteins, NS1, NS2A, NS2B, NS3, NS4A, NS4B, and NS5 (Sharma et al., 2021). The C protein forms the viral nucleocapsid together with viral RNA, whereas the M protein is generated by proteolytic cleavage of precursor membrane protein (prM) during virion maturation, thereby enabling infectivity (Poonsiri et al., 2019). The E glycoprotein plays a central role in viral attachment and membrane fusion and is the major target of neutralizing antibodies. Structural studies of the JEV E protein have identified determinants associated with virion stability, neurovirulence, and receptor interaction (Luca et al., 2012).

JEV exhibits broad tropism and can infect a wide range of hosts, including mammals, birds, ticks, mosquitoes, and other arthropods, as well as diverse tissues and cell types. Like most flaviviruses, JEV mainly enters host cells through clathrin-mediated endocytosis (Carro and Cherry, 2020). Following entry, changes in lipid composition and protein-protein interactions are thought to initiate viral biogenesis. Viral non-structural proteins and viral double-stranded RNA, together with host factors, contribute to the assembly of the JEV replication complex on the endoplasmic reticulum (ER) membrane (Arakawa and Morita, 2019). Immature virions are assembled in the ER, where the viral RNA genome is packaged by the C protein to form the nucleocapsid, which is then enclosed by the E and prM proteins. During transport through the trans-Golgi network, furin-mediated cleavage of prM to M is essential for virion maturation (Li et al., 2008). Although increasing evidence has improved our understanding of viral components involved in JEV entry and replication, the host cell factors participating in these processes remain poorly characterized (Banerjee et al., 2019; Cao et al., 2019; Chiu et al., 2018; Du et al., 2025; Yuan et al., 2024). Elucidating virus-host interactions and the molecular mechanisms underlying JEV transmission will facilitate the identification of antiviral targets for prophylactic and therapeutic development.

In the present study, attenuated JEV SA14-14-2 was used to infect porcine kidney cells (PK15). To identify host proteins interacting with the JEV E protein during infection, immunoprecipitation (IP) combined with LC-MS/MS was performed. Our results showed that host protein annexin A2 (ANXA2) was closely associated with JEV infection and RNA replication. ANXA2 is a member of the annexin family and has been implicated in the organization of specialized membrane microdomains, vesicle budding, membrane fusion, endocytosis, endosomal transport, and membrane repair (Seo and Svenningsson, 2020). To investigate the role of ANXA2 in JEV infection, we generated ANXA2 knockout (KO) PK15 cells using CRISPR/Cas9. We found that ANXA2 deficiency significantly reduced JEV RNA replication and progeny virus titers compared with wild-type (WT) PK15 cells. In addition, fluorescence recovery after photobleaching (FRAP) analysis suggested that ANXA2 contributes to JEV endocytosis and intracellular transport. Together, these findings identify ANXA2 as a key host factor that promotes JEV replication and provide new mechanistic insight into JEV-host interactions, suggesting a potential target for antiviral intervention.

## 2 Materials and methods

### 2.1 Cells and viruses

Porcine Kidney cells (PK15), human embryonic kidney 293T cells (HEK-293T) and Baby hamster kidney cells (BHK-21) present in this study were obtained from Shanghai Zhong Qiao Xin Zhou biotechnology Co., Ltd (China). All above-mentioned media were supplemented with 10% heat-inactivated 10% fetal bovine serum (FBS; Sigma-Aldrich, Merck, Germany) and 1% relevant antibiotics (100 ug/mL streptomycin and 100 U/mL penicillin). All the cells were cultured in minimum essential medium (MEM; Gibco, Grand Island, NY, USA) supplemented with sodium pyruvate, non-essential amino acids (NEAA), and L-glutamine. All the cell line were maintained at 37 °C in a humidified atmosphere containing 5% CO_2_ and passaged every 2–3 days using 0.25% trypsin.

The attenuated JEV strain SA14-14-2 was used in this study. Viral stocks were prepared by infecting PK15 cell monolayers at a multiplicity of infection (MOI) of 0.01–1 when cells reached approximately 80% confluence. Viral adsorption lasts for 2 h; after adsorption, viral inoculum is discarded, cells are washed thoroughly three times with PBS, and fresh maintenance medium is replenished. The time point right after washing is defined as 0 hpi Cell culture supernatants and infected cells lysates were collected at 24–72 h post infection (hpi). Virus-containing supernatants were concentrated by ultracentrifugation at 80,000 × g for 2 h at 4 °C. The pellets were resuspended in phosphate-buffered saline (PBS) and stored at −80 °C until use.

### 2.2 Antibodies and reagents

The primary antibodies used for western blotting, IP, and immunofluorescence were as follows: Annexin A2 polyclonal antibody (Proteintech, 11256-1-AP), GAPDH monoclonal antibody (Proteintech, 60004-1-Ig), Beta Tubulin monoclonal antibody (Proteintech, 66240-1-Ig), and JEV E glycoprotein antibody (Abcam, ab41671), CLTC monoclonal antibody (Proteintech, 66487-1-lg), PI3K/AKT signaling pathway antibodyies used Rabbit recombinant monoclonal anti-AKT1+AKT2+AKT3 antibody (Abcam, ab179463), Rabbit recombinant monoclonal anti-AKT1(pS473)+AKT2(pS474)+AKT3(pS472) antibody (Abcam, ab192623). Secondary antibodies included HRP-conjugated goat anti-rabbit recombinant antibody (Proteintech, RGAR001), HRP-conjugated goat anti-mouse recombinant antibody (Proteintech, RGAM011), iFluor 594-conjugated goat anti-mouse IgG (HUABIO, HA1126), and iFluor 488-conjugated goat anti-rabbit IgG (HUABIO, HA1121). Additional reagents included DiD perchlorate (MCE, HY-D1028) and the lipophilic live-cell fluorescent dye PKH26 (Umibio, UR52302).

### 2.3 IP and co-IP assay

PK15 cells were seeded in culture plates and infected with JEV SA14-14-2 at an MOI of 1. IP experiments were performed using the TAKARA Capturem™ IP & Co-IP Kit (Takara/Clontech, 635721). At 24 hpi, cells were washed three times with ice-cold PBS and lysed in IP lysis buffer (Takara) on ice for 1 h. The lysates were centrifuged at 14,000 rpm for 10 min at 4 °C. Supernatants were incubated with 0.5 μg of JEV E glycoprotein antibody (Abcam, ab41671) for 1 h at 4 °C and then loaded into spin columns containing Capturem Protein A membranes to capture antibody-protein complexes. The columns were centrifuged at 1,000 × g for 1 min at room temperature and washed several times according to the manufacturer's instructions. Bound proteins were eluted with 30–50 μL elution buffer and neutralized immediately. Eluted IP samples and corresponding flow-through fractions were collected for subsequent SDS-PAGE analysis.

### 2.4 LC-MS/MS and proteomic analysis

SDS was added to each sample to a final concentration of 1%. Samples were heated at 95 °C for 5 min and centrifuged. Proteins in the supernatant were precipitated by trichloroacetic acid (TCA) and resuspended in reconstitution buffer containing 8 M urea and 100 mM Tris-HCl (pH 8.5). Protein concentration was determined using the bicinchoninic acid (BCA) assay. Reduction and alkylation were performed using tris(2-carboxyethyl)phosphine (TCEP) and chloroacetamide (CAA) at 37 °C for 1 h. Proteins were digested with trypsin at an enzyme-to-protein ratio of 1:50 (w/w) overnight at 37 °C. The resulting peptides were purified using a self-prepared SDB-RPS desalting column, vacuum-dried, and stored at −20 °C until analysis.

Peptide samples were analyzed using an UltiMate 3000 RSLCnano system coupled online to a Q Exactive HF mass spectrometer via a Nanospray Flex ion source (Thermo Fisher Scientific). Samples were loaded onto a C18 Trap column (75 μm × 2 cm, 3 μm particle size, 100 Å pore size; Thermo Fisher Scientific) and separated on a reversed-phase C18 analytical column packed in-house with ReproSil-Pur C18-AQ resin (75 μm × 25 cm, 1.9 μm particle size, 100 Å pore size) and continuous detection for 60 min each sample. Mass spectrometry was performed in data-dependent acquisition (DDA) top 20 mode with a full scan range of 350–1,500 m/z. The AGC target for full MS scans was 3 × 10^∧^6 charges, with a maximum injection time of 30 ms and a resolution of 60,000 at m/z 200.

Raw MS data were analyzed using MaxQuant software (version 2.2.0.0) with the Andromeda search engine. Spectra were searched against the Sus scrofa protein database downloaded from UniProt (2024-01-05). Carbamidomethylation (Cys) was set as a fixed modification, whereas oxidation (Met), acetylation (protein N-terminus), and deamidation (Asn/Gln) were set as variable modifications. Trypsin/P specificity was used with a maximum of two missed cleavages. The precursor mass tolerances for the first search and main search were set to 20 ppm and 4.5 ppm, respectively, and the fragment mass tolerance was set to 20 ppm. Protein groups supported only by non-unique peptides were merged by MaxQuant. Search results were filtered at a false discovery rate (FDR) of 1% at both the peptide and protein levels. Reverse hits, potential contaminants, and proteins identified only by site were removed. Downstream bioinformatics analyses were performed in R. Functional annotation was carried out using Gene Ontology (GO), the Kyoto Encyclopedia of Genes and Genomes (KEGG), EggNOG, Pfam, and UniProt subcellular localization databases.

Protein-protein interaction (PPI) analysis of differentially expressed proteins was performed using the STRING database (https://cn.string-db.org/). The top 50 differentially expressed proteins identified in infected cells, based on MaxQuant scores, were selected for PPI analysis. GO and KEGG enrichment analyses were performed using STRING v12.0 with Sus scrofa as the background organism. A minimum required interaction score of 0.4 was used, and the FDR threshold was set at 5%. Results were exported as TSV files, and figures were generated using the Bioinformatics online platform (www.bioinformatics.com.cn).

### 2.5 Immunofluorescence and confocal microscopy

To detect JEV infection and assess co-localization between ANXA2 and the JEV E protein, immunofluorescence staining was performed followed by confocal microscopy. Approximately 1 × 10^∧^4 cells were seeded into 35-mm glass-bottom dishes (Thermo Scientific Nunc, 150680) and cultured for 24 h at 37 °C in 5% CO_2_. PK15 cells were infected with JEV SA14-14-2 at an MOI of 1 at approximately 60% confluence. At 48 hpi, cells were washed three times with PBS, fixed with 4% paraformaldehyde for 30 min, and permeabilized with 0.1% Triton X-100 for 10 min at room temperature. Cells were blocked overnight at 4 °C with 5% BSA and then incubated with primary antibodies against ANXA2 (1:500) or JEV E glycoprotein (1:20) overnight at 4 °C in 5% BSA. After five washes with PBST (PBS containing 0.2% Tween 20), cells were incubated with the corresponding fluorescent secondary antibodies for 2 h in the dark at room temperature. Finally, DAPI mounting medium was added to each dish. Images were captured using a ZEISS LSM 710 laser confocal microscope.

Image analysis and quantification of infected cells were performed using Fiji/ImageJ software (version 1.53f51). For two-color images, channels were separated in Fiji and converted to 8-bit grayscale. Thresholds were adjusted manually before the channels were merged into binary images. The DAPI channel was used to count total cells using the Analyze Particles function with a size filter of 50–500 pixels. Fluorescent signals were quantified by overlaying the target channel with the DAPI channel using Image Calculator, and adjacent signals were merged to calculate the proportion of infected cells.

### 2.6 Western-blot analysis

Cells were washed three times with ice-cold PBS and lysed in radioimmunoprecipitation assay (RIPA) buffer (epizyme, PC101) containing protease inhibitor and phosphatase inhibitor (10uM Sorlabio). Lysates were incubated on ice for 1 h and centrifuged at 14,000 × g for 10 min at 4 °C to remove insoluble material. Protein concentrations were determined using the BCA assay (TaKaRa, T9300A). For western blotting, 30–50 μg of protein mixed with loading buffer (epizyme, LT103) was heated at 100 °C and separated on 10% SDS-PAGE gels. Proteins were transferred to 0.45 μm PVDF membranes (Millipore, 1.15546), which were blocked with 5% non-fat milk for 2 h at room temperature and incubated overnight at 4 °C with the appropriate primary antibodies. Membranes were washed with Phosphate Buffered Saline with Tween 20 (PBST) three times and then incubated with HRP-conjugated secondary antibodies for 1 h at room temperature. Protein bands were detected using enhanced chemiluminescence (ECL) reagents (Epizyme, Omni-ECL Universal Chemiluminescence Detection Kit, SQ203). After exposure, store the PVDF membrane at 4 °C in PBST and incubated with Western Blot Fast Stripping Buffer (Epizyme, PS107) for 20–30 min at room temperature to strip off all antibodies bound to the membrane. After washing with PBST, the PVDF membrane was blocked again and incubated other antibodies for detection.

### 2.7 Quantification of viral RNA and host gene expression by RT-qPCR

Total RNA was extracted from PK15 cells using RNAiso Plus reagent (TaKaRa, T9108), and RNA from culture supernatants of JEV-infected cells was extracted using the TaKaRa MiniBEST Viral RNA/DNA Extraction Kit (TaKaRa, version 5.0, code no. 9766) according to the manufacturer's instructions. Mock-infected cells and supernatants were processed in parallel as negative controls. Extracted RNA was reverse-transcribed into cDNA using the PrimeScript™ RT Reagent Kit with gDNA Eraser (Perfect Real Time; TaKaRa, RR047Q). RT-qPCR was performed using TB Green^®^ Premix Ex Taq™ (Perfect Real Time; TaKaRa, RR0091A).

Each 20 μL PCR mixture contained 10 μL TB Green Premix Ex Taq (2 × ), 0.4 μL ROX Reference Dye II, 0.6 μL each of forward and reverse primers (10 μM), 6.4 μL H2O, and 2 μL cDNA template (10 ng). Reactions were performed in triplicate biological replicates on a QuantStudio™ 5 Real-Time PCR System (Applied Biosystems, USA) under the following conditions: 50 °C for 2 min, 95 °C for 2 min, followed by 40 cycles of 95 °C for 15 s and 60 °C for 30 s. Because an effective internal reference gene is not available in culture supernatants, viral RNA copy numbers were determined from RNA extracted from equal volumes of infected-cell supernatants. A standard curve was generated using serial dilutions of a recombinant plasmid containing the JEV 3′-UTR target sequence. β-actin was reference gene to normalize cDNA input for cellular gene expression analysis. Primer sequences are listed in Supplementary Table 1.

### 2.8 ANXA2 knockout in PK15 cells using CRISPR/Cas9

Two pairs of gRNAs targeting the exon region of the ANXA2 gene (GenBank Gene ID: 406192) were designed using the CRISPR Design online tool (https://portals.broadinstitute.org/gppx/crispick/public) (shown as Supplementary Table 2). Oligonucleotides corresponding to the gRNA sequences and their reverse complements were synthesized, annealed using T4 PNK (NEB) at 37 °C for 30 min followed by 95 °C for 5 min, and cooled to room temperature. The pLenti-CRISPR/Cas9-2A-puro vector was digested with BsmBI (NEB), and the recombinant pLenti-CRISPR/Cas9-gRNA-ANXA2 plasmid was generated by ligation of the annealed gRNA duplexes into the linearized vector using T4 DNA ligase (NEB).

HEK293T cells were co-transfected with pLenti-CRISPR/Cas9-gRNA-ANXA2, psPAX2, and VSV-G plasmids to produce lentiviral particles. Before transfection, cells were washed twice with RNase-free PBS to remove FBS. ANXA2-targeting or control siRNA (10 nM) and Lipofectamine 2000 (Invitrogen) were separately diluted in Opti-MEM. After incubation for 20 min at room temperature, the complexes were added to the cells, which were then cultured for 6 h at 37 °C in 5% CO_2_. The supernatants were collected and used to infect PK15 cells for 1 h at 37 °C. The medium was then replaced with MEM containing 2% FBS, and cells were cultured for an additional 48 h. EGFP expression in infected PK15 cells was observed using an inverted fluorescence microscope (Leica). EGFP-positive cells were further sorted using a BD FACSAria III flow cytometer. More than 10^∧^5 EGFP-positive cells were collected, and the proportion of EGFP-positive cells was confirmed to exceed 98%. Sorted cells were then subjected to limiting dilution cloning in 96-well plates containing 100 μL complete MEM per well. After 7 days of culture at 37 °C in 5% CO_2_, genomic DNA from positive monoclonal colonies was extracted, and the target locus was amplified by PCR using TaKaRa PrimeSTAR^®^ Max DNA Polymerase under the following cycling conditions: 95 °C for 2 min; 35 cycles of 95 °C for 20 s, 55 °C for 20 s, and 72 °C for 45 s; and a final extension at 72 °C for 5 min. PCR products were subjected to Sanger sequencing to identify indel mutations. Potential off-target sites were also amplified and sequenced.

### 2.9 Cell viability and flow cytometry

Cell viability of ANXA2 knockout PK15 cells (ANXA2-KO) and wild-type PK15 cells (WT) was assessed using the Cell Counting Kit-8 (CCK-8; MCE, China, HY-K0301). Cells were seeded in 96-well plates at 5 × 10^∧^3 cells per well in 100 μL MEM containing 10% FBS. At least five replicate wells were used for each group or condition. After 24 h of culture, 10 μL CCK-8 solution was added to each well, and the plates were incubated for 2 h at 37 °C. Absorbance was measured at 450 nm using a Bio-Rad microplate reader (Model 680; Bio-Rad Laboratories, Hercules, CA, USA).

For cell cycle and apoptosis analysis, ANXA2-KO and WT PK15 cells were detached using 0.25% trypsin without EDTA, centrifuged at 300 × g for 5 min at room temperature, and resuspended in 500 μL PBS. Apoptosis and cell cycle distribution were analyzed using a Cell Cycle and Apoptosis Analysis Kit (Beyotime, C1052) according to the manufacturer's instructions. Cells were fixed overnight in 70% ethanol at 4 °C, washed three times with cold PBS, and resuspended in 500 μL binding buffer containing 25 μL propidium iodide (PI). Cells were incubated for 30 min at 37 °C in the dark and analyzed using a FACSCanto Plus flow cytometer. Each experiment included three biological replicates. Data were analyzed using FlowJo software (version 10).

### 2.10 CME inhibitor and knockout internalization assay

Dynasore (MCE, China, HY-15304) acts as a potent dynamin inhibitor to block clathrin-mediated endocytosis (CME). To identify a non-cytotoxic and effective concentration for PK15 porcine kidney epithelial cells, a concentration gradient of Dynasore (0, 40, 80, and 100 μM) was screened via CCK-8 assay. The 80 μM concentration was selected for subsequent experiments, for long-term treatment over 12 h in serum-free medium to guarantee maximal inhibitory efficiency. An equal volume of dimethyl sulfoxide (DMSO) was added to control groups to eliminate solvent interference.

Further experiments using transferrin (Tf), a canonical ligand for the CME pathway, were performed to verify the regulatory effect of ANXA2 knockout on clathrin-mediated endocytosis. Dynasore acts as a potent dynamin inhibitor to block clathrin-mediated endocytosis (CME). Cy3-conjugated transferrin (Cy3-Tf, MCE, China, HY-D2426) was utilized as a classic tracer to quantify cellular intrinsic CME activity. Wild-type and ANXA2-knockout PK15 cells were treated with 80 μM Dynasore for 12 h, followed by medium replacement. Cells were incubated with serum-free medium containing 80 μg/mL Cy3-Tf at room temperature in the dark for 30 min. After ligand binding, the 10%FBS complete medium was added the cells at 37 °C in the dark for incubated 30 min to initiate endocytic internalization. Cells were washed three times with PBS, digested by trypsin, resuspended in PBS and filtered. Flow cytometry was performed using the PE-Cy3 channel to measure the mean fluorescence intensity (MFI) of internalized Cy3-Tf.

### 2.11 TCID50 titration and virus growth curve

Virus titers were determined by TCID50 assay according to the Reed–Muench method (Lei et al., 2021). Briefly, BHK-21 cells were seeded in 96-well plates to form a confluent monolayer. Virus-containing supernatants were thawed and serially diluted 10-fold in MEM containing 2% FBS over a dilution range of 10^∧^2 to 10^∧^8. Each dilution was inoculated into 4–8 replicate wells (100 μL per well). Wells receiving PBS served as negative controls. Cells were cultured at 37 °C in 5% CO_2_ for 48 h, after which cytopathic effects were recorded and TCID50 values were calculated using the Reed–Muench method.

For multi-step growth curve analysis, BHK-21 cell monolayers were infected with JEV SA14-14-2 at an MOI of 0.1 and incubated for 2 h at 37 °C. The inoculum was then removed, cells were washed twice with PBS, and fresh MEM containing 2% FBS was added. Supernatants were collected at 6, 12, 18, 24, 36, 48, and 60 hpi. Viral titers at each time point were determined by TCID50 assay.

### 2.12 Live-cell imaging and FRAP

PK15 cells were infected with JEV at an MOI of 1 as described above, and culture supernatants were collected at 72 hpi. Supernatants were filtered through a 0.22 μm filter (Millipore Millex™-GP, 0.22 μm, 33 mm, hydrophilic polyethersulfone membrane) to remove cell debris and large particles. The filtrate was centrifuged at 14,000 rpm for 30 min at 4 °C, transferred to ultracentrifuge tubes, and further centrifuged at 120,000 × g for 2 h at 4 °C using a HITACHI CP100NX ultracentrifuge. After centrifugation, the pellet was resuspended in 200 μL PBS and stored at −80 °C.

The lipophilic fluorescent probe DiD was used to label virus particles. DiD-stained virus was incubated with 1 μM dye for 30 min at room temperature and then purified by ultracentrifugation to remove excess dye. For live-cell imaging, 35-mm glass-bottom confocal dishes (BioSharp) coated with 0.4% gelatin were used. DiD-labeled JEV particles were added to ANXA2-KO or WT PK15 cells at an MOI of 10 and incubated for 24 h. Fluorescence images were acquired using a ZEISS LSM 710 confocal microscope equipped with a Plan-Apochromat 63 × /1.40 NA Oil DIC M27 objective. FRAP analysis was performed using ZEN Blue software. For FRAP, regions of interest with a diameter of 20 μm within DiD-positive structures were bleached at 100% laser power for 0.2 s per cycle, repeated 20 times using a 633 nm laser. Fluorescence recovery was recorded every 5 s for up to 10 min. Fluorescence intensity was also measured in an unbleached reference area, and a background area without fluorescence was used for correction. Relative fluorescence intensity (RFI) was calculated as previously described (Chen and Huang, 2001).

### 2.13 Statistical analysis

Data are presented as mean ± standard deviation (SD). Homogeneity of variance was assessed using the F-test for pairwise comparisons or the Brown-Forsythe test for multiple-group comparisons. The Shapiro-Wilk test was used to assess normality. Student's *t*-test and Welch's *t*-test were applied to normally and non-normally distributed data, respectively, for two-group comparisons. One-way or two-way ANOVA was used for comparisons involving one or two factors, respectively. *P*-values were adjusted for multiple comparisons where appropriate. Statistical analyses were performed using GraphPad Prism 8 (GraphPad Software, San Diego, CA, USA). The *P*-value < 0.05 was considered statistically significant. ^*^P < 0.05, ^**^P < 0.01, ^***^P < 0.001; ns, not significant.

## 3 Results

### 3.1 RNA replication and viral production of the attenuated JEV strain SA14-14-2 in PK15 cells

**Figure 1:**
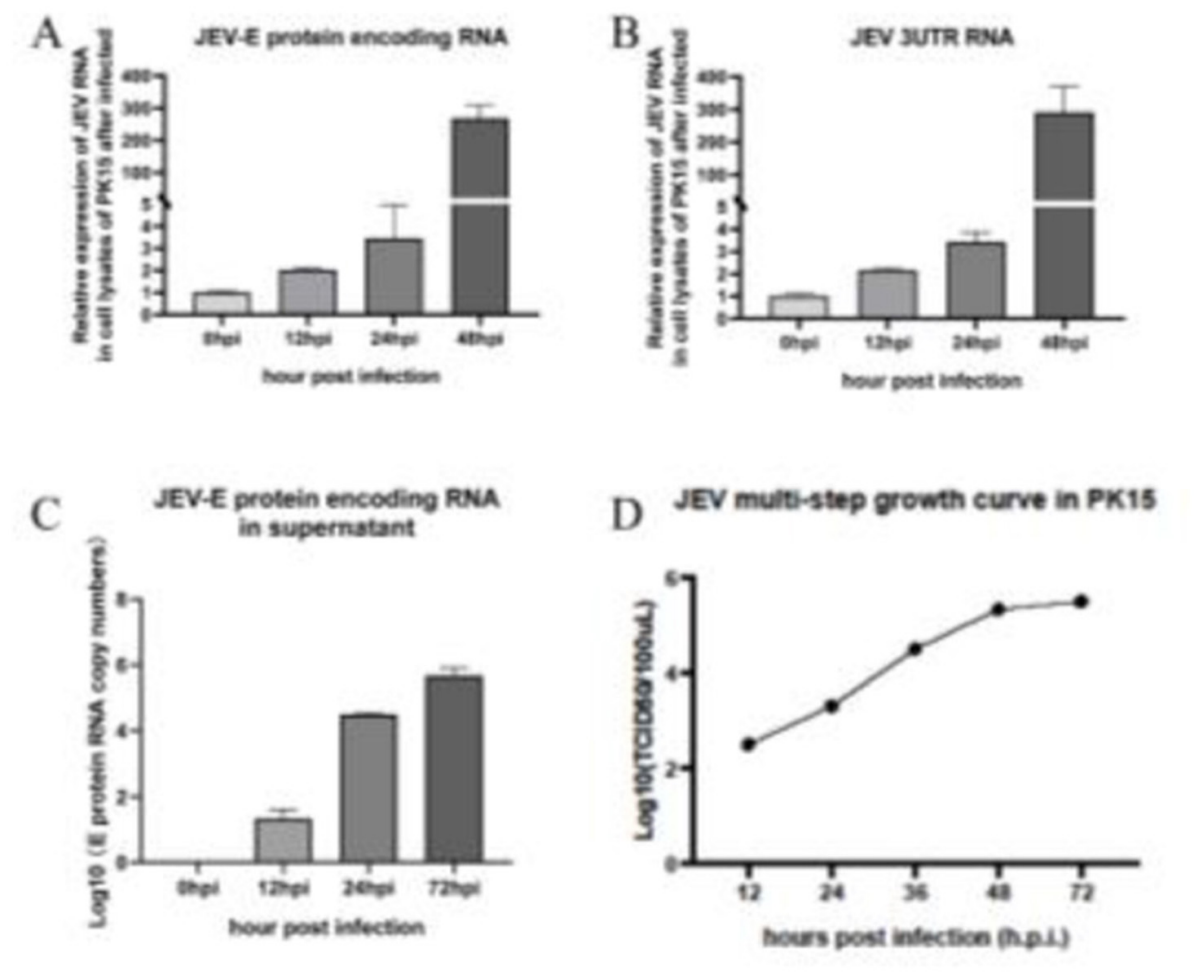
RNA Replication kinetics and viral production of attenuated JEV SA14-14-2 in PK15 cells. **(A)**: Relative levels of JEV E protein encoding RNA was measured from cell lysates at different time points after infection, determined by qRT-PCR. **(B)** Relative levels of JEV 3′UTR RNA in PK15 cell lysates at different time points after infection, determined by qRT-PCR. **(C)** The Viral E protein encoding RNA was measured from cells culture supermatant by qRT-PCR. **(D)** Viral titers in culture supernatants collected from JEV-infected PK15 cells at 24–72 hpi, determined by TCID50 assay. Data are shown as mean ± SD from three independent experiments. Four technical replicates were included for each sample.

We first evaluated the replication capacity of the attenuated JEV strain SA14-14-2 in PK15 cells. Quantification of viral RNA over time showed that JEV RNA was detectable at an early stage of infection and increased progressively in both cell lysates and culture supernatants (Figures 1A–C). Both two of viral RNA (E protein encoding RNA and 3′UTR RNA) was detected in cell lysates as early as 12 hpi and in supernatants at 24 hpi, respectively. To determine whether infected PK15 cells produced infectious progeny virus, culture supernatants collected at different time points of infection were titrated on BHK cells. The results showed that infectious virus was readily detected in the supernatants, and viral titers increased in a time-dependent manner (Figure 1D). The titer in the supernatant of PK15 cells reached a peak of approximately 10^∧^5 TCID50/mL at 36 hpi. These results indicate that the attenuated JEV strain SA14-14-2 efficiently infects PK15 cells and that 24–48 hpi represents a suitable window for subsequent analyses.

### 3.2 Identification of host factors associated with JEV infection by IP-LC-MS/MS

To investigate host proteins associated with JEV infection and replication, we performed IP combined with LC-MS/MS to isolate protein complexes associated with the JEV E protein from infected PK15 cells. PK15 cells were infected with JEV at an MOI of 1, and cell lysates were collected at 24 hpi. Proteins were immunoprecipitated using an antibody against the JEV E protein. Nonspecific IgG and uninfected cells processed with the same antibody were used as negative controls. The immunoprecipitated samples were separated by column chromatography and visualized by Coomassie Brilliant Blue staining in SDS-PAGE (Supplementary Figure 1E).

**Figure 2:**
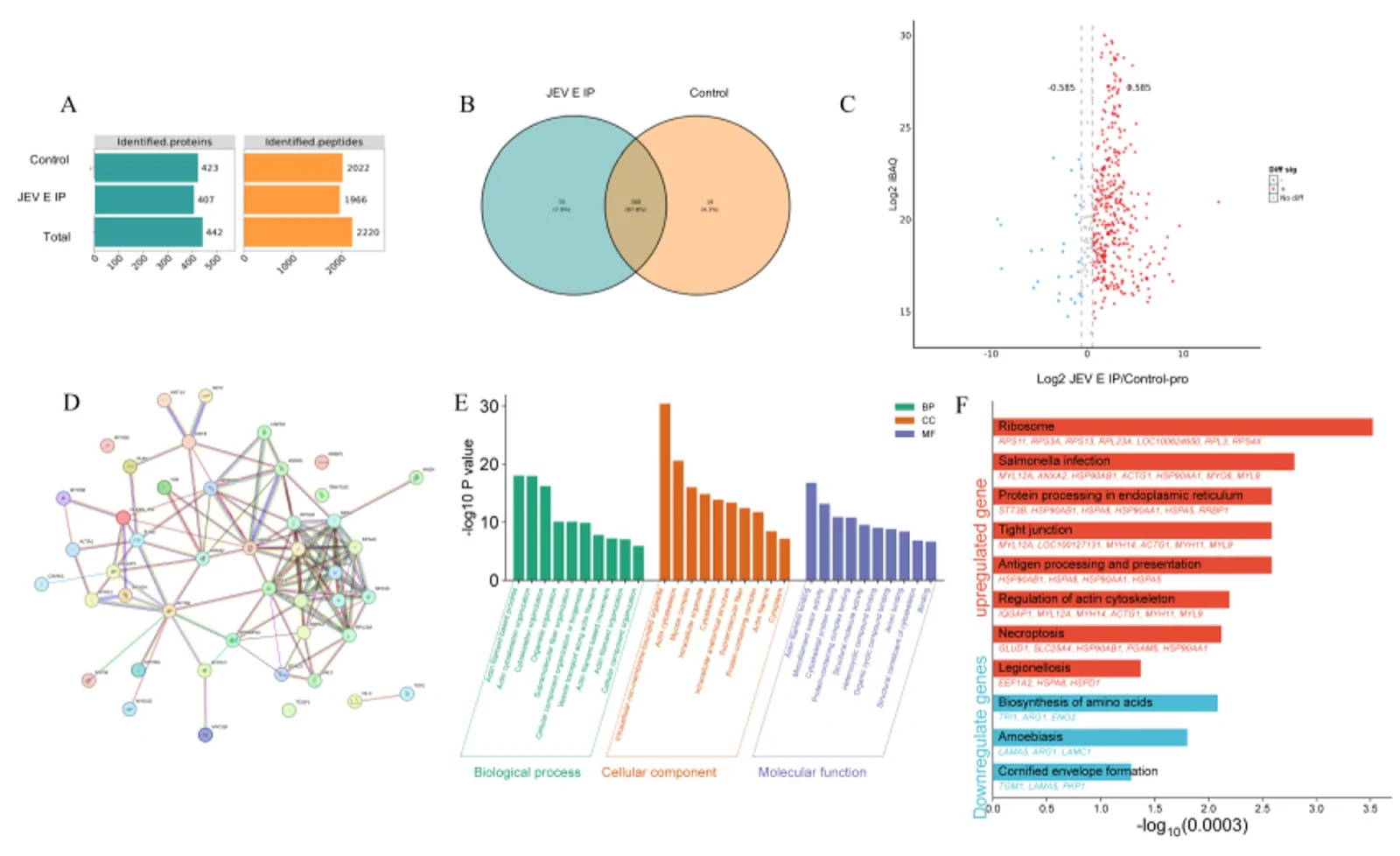
Identification of host factors associated with JEV infection by LC-MS/MS. **(A)** LC-MS/MS identification of proteins and peptides from PK15 cells. JEV E IP was the immunoprecipitated samples obtained from PK15 cells harvested 24 h post-infection. Control was the immunoprecipitated samples obtained from Uninfected cells processed under the same conditions. **(B)** Venn diagram illustrating the intersection of proteins identified from JEV E IP group and control group. **(C)** The ratio volcano of differentially expressed proteins between JEV E IP and Control. **(D)** Protein-protein interaction network of intersecting proteins generated using the STRING database. **(E)** GO enrichment analysis of the identified proteins. Biological process (BP), cellular component (CC), and molecular function (MF) categories are shown in green, sienna, and blue, respectively. **(F)** KEGG pathway enrichment analysis of the identified proteins. Upregulated genes are shown in red and downregulated genes in cyan; pathway names are shown in black and gene names in red.

After tryptic digestion and LC-MS/MS analysis, a total of 423 proteins were identified across the infected and control groups. Of these, 388 proteins were shared by both groups, whereas 35 proteins were detected only in the JEV-infected pulldown samples (Figure 2A). To identify candidate interactors, we applied a threshold of |Log2 fold change| > 1 and an FDR-adjusted *P*-value < 0.05. Under these criteria, 322 proteins were significantly enriched in the infected pulldown samples, whereas 27 proteins showed significant reduction in abundance compared with the control pulldown samples (Figures 2B, C).

In order to understand the functions and interactions between differentially expressed host proteins identified in JEV infection, the top 50 upregulated proteins ranked by MaxQuant score were selected for protein-protein interaction (PPI) analysis using the STRING database. The resulting network contained 46 nodes with medium-confidence interactions (Figure 2D). Topological analysis identified several hub proteins with higher connectivity, including HSP90AB1, ANXA2, ACTG1, HSPA8, RPL3, RPL23A, RPS11, and RPS18. GO and KEGG enrichment analysis suggested that these proteins were mainly associated with actin filament-based processes, cytoskeletal organization, intracellular non-membrane-bounded organelles, actin cytoskeleton, and myosin complexes. The enriched molecular functions included actin filament binding, microfilament motor activity, and cytoskeletal protein binding (Figures 2E, F). These findings suggest that the host proteins associated with JEV E protein are mainly involved in cytoskeletal remodeling, intracellular transport, translation-related processes, and other pathways potentially important for JEV infection.

### 3.3 ANXA2 is associated with JEV infection and affects early viral RNA replication

**Figure 3:**
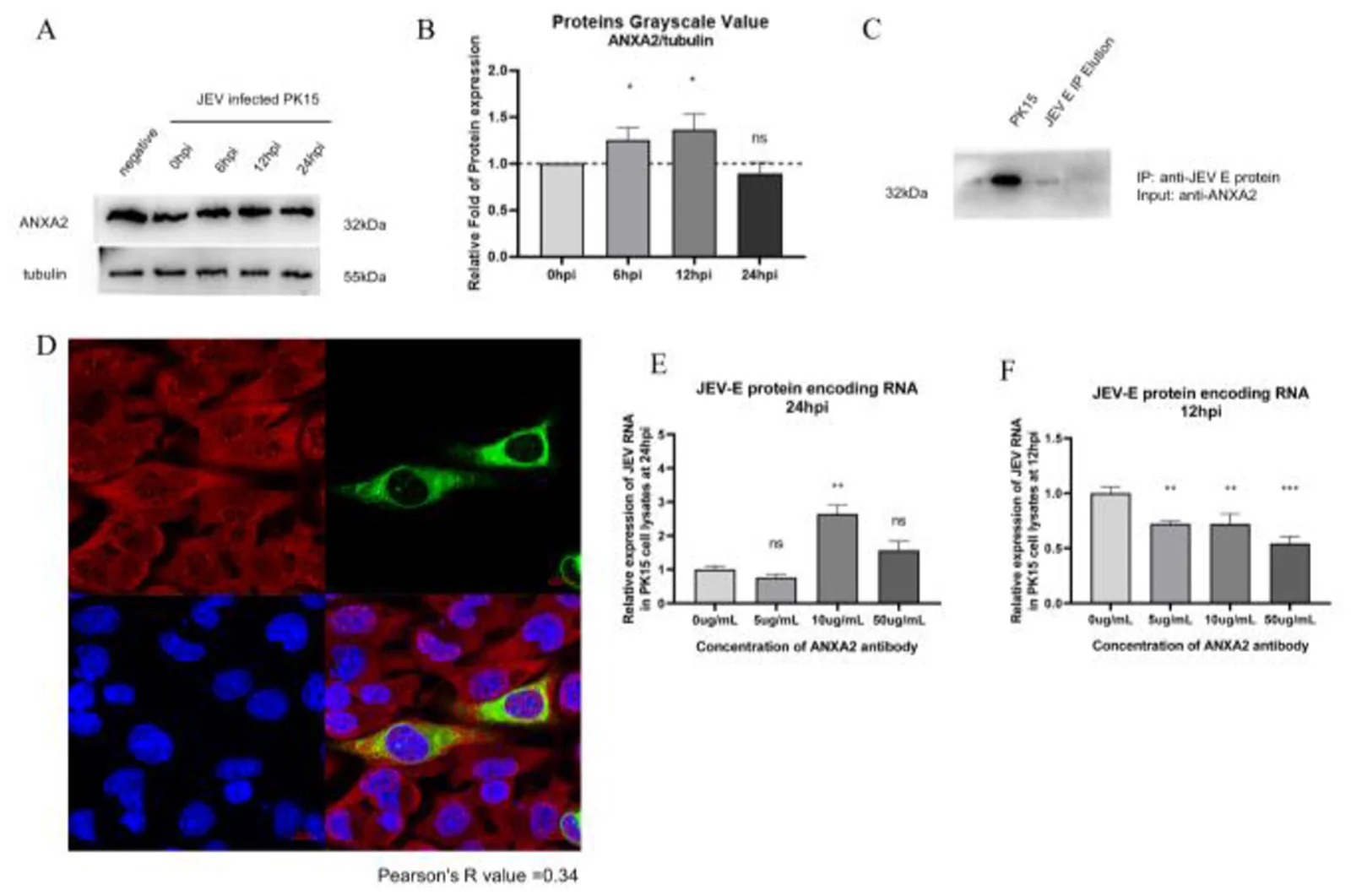
ANXA2 is associated with JEV infection and affects early JEV RNA replication. **(A)** Western blot analysis of ANXA2 expression in PK15 cells at different time points after JEV infection. **(B)** Grayscale quantification of the ANXA2 band shown in panel **(A)**. **(C)** Co-immunoprecipitation of ANXA2 and JEV E protein using anti-JEV E monoclonal antibody and anti-ANXA2 polyclonal antibody. **(D)** Confocal immunofluorescence analysis of co-localization between JEV E protein and ANXA2 in infected PK15 cells. JEV E protein was detected with iFluor 488-conjugated goat anti-rabbit IgG (green), ANXA2 was detected with iFluor 594-conjugated goat anti-rabbit IgG (red), and nuclei were counterstained with DAPI (blue). Pearson's correlation coefficient was calculated using Fiji/ImageJ. Scale bar, 10 μm. **(E)** Relative levels of JEV RNAs in PK15 cell lysates after treatment with different concentrations of anti-ANXA2 polyclonal antibody at 24 hpi, determined by qRT-PCR. **(F)** Relative levels of JEV RNA in PK15 cell lysates after treatment with different concentrations of anti-ANXA2 polyclonal antibody at 12 hpi, determined by qRT-PCR. Data are shown as mean ± SD from three independent experiments. *P < 0.05; **P < 0.01; ***P < 0.001; ns, not significant.

In order to screen membrane-associated host proteins that participate in viral binding, cellular entry and receptor-mediated internalization. We excluded the proteins which the functional classification revealed t in intracellular metabolism, nuclear transport and ribosomal biogenesis so them and focused on annexin family proteins linked to cytoskeleton and clathrin-mediated vesicle trafficking. Based on the above screening criteria and prior research basis, ANXA2, which was significantly enriched and upregulated in JEV E protein co-IP products, was selected as the core candidate host factor for follow-up functional verification. Among the candidate host proteins identified by LC-MS/MS, we focused on annexin A2 (ANXA2) because it showed more than a two-fold increase in abundance in the infected pulldown samples (Log2 fold change = 1.8) and exhibited a high interaction score in the PPI network. Western blot analysis further showed that ANXA2 expression was increased in PK15 cells at 6–12 hpi following early stage of JEV infection (Figures 3A, B). To validate the interaction between ANXA2 and the JEV E protein, co-immunoprecipitation (Co-IP) assays were performed using total protein extracted from JEV-infected PK15 cells. The results confirmed that ANXA2 was associated with the JEV E protein (Figure 3C and Supplementary Figure 1D). However, comparison of the PK15 IP Loading flow-through sample and JEV E IP elution fractions indicated that most cellular ANXA2 remained in the flow-through, and only a small fraction of ANXA2 was interacted with the IP antibodies and recovered in the pulldown fraction, suggesting that the interaction between ANXA2 and the JEV E protein was relatively weak or transient. This may reflect stage-specific or spatially restricted protein interactions during viral infection. Consistent with this interpretation, confocal immunofluorescence analysis did not reveal a strong co-localization signal (Pearson's R value = 0.34) between ANXA2 and the JEV E protein in infected PK15 cells at 12hpi (Figure 3D and Supplementary Figures 1A–C).

To further assess whether ANXA2 contributes to JEV infection, PK15 cells were incubated with anti-ANXA2 antibodies at different concentrations during infection, and viral RNA replication was quantified by RT-qPCR. ANXA2 antibody treatment inhibited JEV RNA replication in a dose-dependent manner, with higher antibody concentrations producing a stronger suppressive effect during the early phase of infection (12hpi) (Figures 3E, F). These results suggest that ANXA2 contributes to early JEV RNA replication and was therefore selected for further investigation.

### 3.4 Generation and characterization of ANXA2 knockout PK15 cells using CRISPR/Cas9

**Figure 4:**
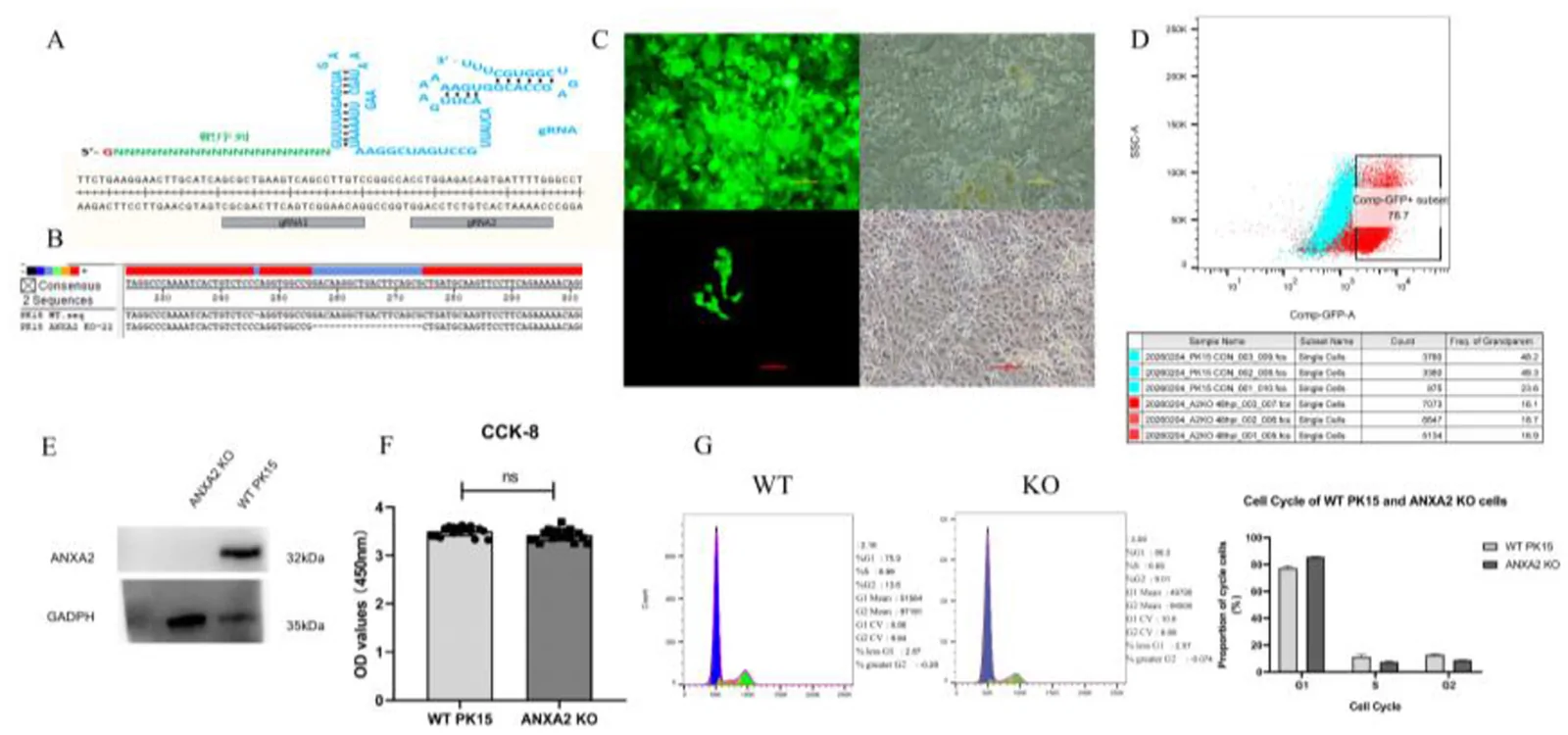
Generation and characterization of ANXA2KO PK15 cells by using CRISPR/Cas9. **(A)** Schematic representation of the sgRNAs target sites and corresponding sequences used in this study. **(B)** Sanger sequencing results of the gRNAs target sites in individual ANXA2-KO PK15 clones. **(C)** EGFP fluorescence in 293T cells 48 h after transfection with lentiviral packaging plasmids (top two images) and the packaged lentiviral infected PK15 cells at 48 hpi (bottom two images). **(D)** EGFP-positive cells analyzed by flow cytometry. Cyan dots represent cells before sorting, and red dots represent sorted cells. **(E)** ANXA2 protein expression in ANXA2-KO and WT PK15 cells, determined by western blot. **(F)** Cell viability of ANXA2-KO and WT PK15 cells, determined by CCK-8 assay. **(G)** Cell cycle of ANXA2-KO and WT PK15 cells, determined by flow cytometry. Data are shown as mean ± SD from three independent experiments. ns, not significant.

To determine the role of ANXA2 in JEV infection more directly, we generated ANXA2 knockout PK15 cell lines using the CRISPR/Cas9 system. Two sgRNAs targeting the exon region of the ANXA2 coding sequence (GenBank Gene ID: 406192) were designed and cloned into the pLenti-CRISPR/Cas9-EGFP vector (Figure 4A). After lentiviral transduction, EGFP-positive PK15 cells were sorted by flow cytometry and further subcloned by limiting dilution. Sanger sequencing of individual clones revealed a 19-bp deletion in the ANXA2 exon, resulting in a frameshift mutation (Figure 4B). Western blot analysis confirmed that ANXA2 protein expression was undetectable in the selected mutant clone compared with wildtype (WT) PK15 cells, indicating successful generation of ANXA2 knockout (ANXA2-KO) cells (Figures 4C–E).

To exclude potential effects of ANXA2 deletion on cell growth, we assessed cell viability and cell cycle distribution. CCK-8 assays showed no significant difference in viability between ANXA2-KO and WT PK15 cells (Figure 4F). In addition, flow cytometric analysis revealed no obvious alteration in cell cycle profile in ANXA2-KO cells compared with WT cells (Figure 4G). These results indicate that ANXA2 knockout did not substantially affect baseline cell viability or cell cycle progression.

### 3.5 ANXA2 knockout suppresses JEV RNA replication and viral production in PK15 cells

**Figure 5:**
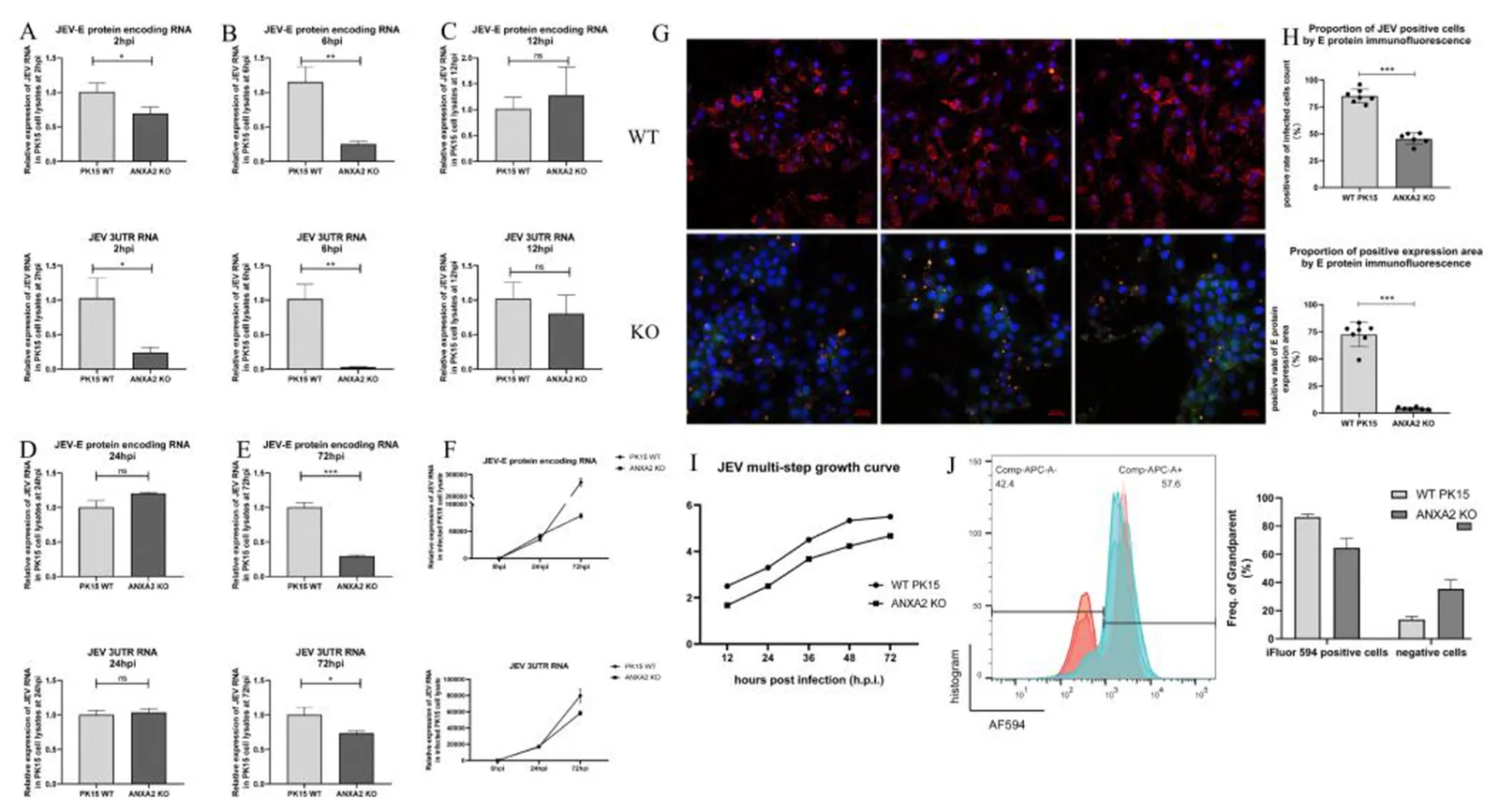
ANXA2 knockout suppresses JEV RNA replication and viral production. **(A)** Relative levels of JEV RNA in WT and ANXA2-KO PK15 at 2 hpi, determined by qRT-PCR. **(B)** Relative levels of JEV RNA in WT and ANXA2-KO PK15 at 6 hpi, determined by qRT-PCR. **(C)** Relative levels of JEV RNA in WT and ANXA2-KO PK15 at 12 hpi, determined by qRT-PCR. **(D)** Relative levels of JEV RNA in WT and ANXA2-KO PK15 at 24 hpi, determined by qRT-PCR. **(E)** Relative levels of JEV RNA in WT and ANXA2-KO PK15 at 72 hpi, determined by qRT-PCR. **(F)** Intracellular JEV RNA accumulation between 24 and 72 hpi in WT and ANXA2-KO PK15 cells, determined by qRT-PCR. **(G)** Indirect immunofluorescence analysis of JEV E protein expression in WT and ANXA2-KO PK15 cells at 48 hpi. JEV E protein was detected with iFluor 594-conjugated goat anti-rabbit IgG (red), nuclei were stained with DAPI (blue), and EGFP indicated ANXA2-KO cells (green). **(H)** Quantification of JEV E protein-positive cells rate and signal area in panel **(G)**. **(I)** Viral titers in culture supernatants from WT and ANXA2-KO PK15 cells infected with JEV, determined by TCID50 assay. Multi-step growth curves are shown. **(J)** Frequency of JEV E protein-positive cells analyzed by flow cytometry. Data are shown as mean ± SD from three independent experiments. *P < 0.05; **P < 0.01; ***P < 0.001; ns, not significant.

To evaluate whether ANXA2 contributes to JEV replication, ANXA2-KO and WT PK15 cells were infected with JEV at an MOI of 1, and the relative expression of viral RNA levels in cell lysates were measured at 2hpi, 6hpi, 12hpi, 24hpi and 72hpi by qRT-PCR. The results showed that both JEV 3UTR RNA and E protein encoding RNA were significantly reduced in ANXA2-KO cells at 2hpi, 6hpi and 72hpi (Figures 5A, B, E). Indicating that ANXA2 Knockout impaired JEV entry and early stage replicated. However, No significant difference in JEV 3UTR or E gene RNA levels was observed between ANXA2-KO and WT cells at 12hpi and 24hpi (Figures 5C, D). In addition, the accumulation of viral RNA between 24 and 72 hpi was slower in ANXA2-KO cells than in WT cells (Figure 5F).

To assess the effect of ANXA2 deficiency on infection efficiency, JEV E protein-positive cells were detected by immunofluorescence and flow cytometry at 48 hpi. Compared with WT cells, ANXA2-KO cells showed a markedly reduced number of E protein-positive cells and a lower fluorescent signal intensity (Figures 5G, H). Flow cytometry further confirmed a lower infection rate in ANXA2-KO cells than in WT cells (Figure 5J). To determine whether ANXA2 knockout affected progeny virus production, supernatants were collected from infected cells and titrated by TCID50 assay. Compared with WT controls, ANXA2-KO cells produced significantly lower viral titers (Figure 5I). Collectively, these findings demonstrate that ANXA2 promotes JEV infection and replication in PK15 cells.

### 3.6 ANXA2 knockout effects the expression of clathrin-mediated endocytosis-related proteins and inhibits the activation of PI3K/Akt signaling pathway in early JEV infection

**Figure 6:**
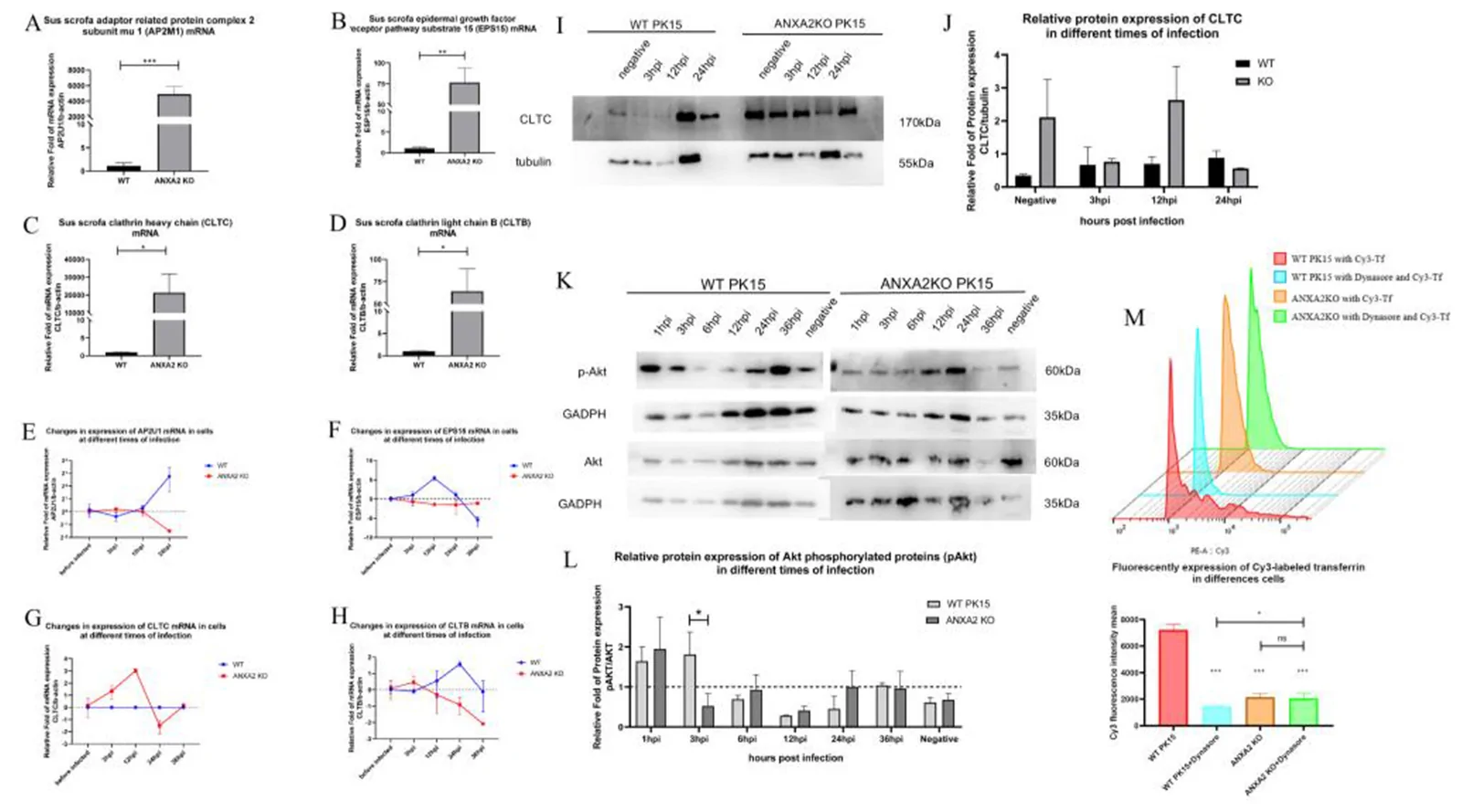
ANXA2 knockout alters clathrin-mediated endocytosis-related gene expression and Akt phosphorylation during early JEV infection. **(A–D)** mRNA levels of endocytosis-related genes, including AP2M1, EPS15, CLTC, and CLTB, in WT and ANXA2-KO PK15 cells. **(E–H)** Time-course analysis of AP2M1, EPS15, CLTC, and CLTB mRNA levels in WT and ANXA2-KO PK15 cells during JEV infection. WT cells are shown in blue and ANXA2-KO cells in red. **(I, J)** Western blot analysis of CLTC protein levels in WT and ANXA2-KO PK15 cells at different time points after JEV infection. **(K, L)** Western blot analysis of p-AKT in total AKT levels in WT and ANXA2-KO PK15 cells at different time points after JEV infection. **(M)** Flow cytometry measures the mean fluorescence intensity (MFI) of internalized Cy3-Tf in WT or ANXA2-KO PK15 cells. Data are shown as mean ± SD from three independent experiments. *P < 0.05; **P < 0.01; ***P < 0.001; ns, not significant. All representative experiment out of three repeats shown as Supplementary Figure.

Based on the known function of ANXA2 in host cell's endosomal trafficking and membrane dynamics, we examined whether ANXA2 affects clathrin-mediated endocytosis during JEV infection. We analyzed the mRNA expression of several endocytosis-related factors, including clathrin heavy chain (CLTC), clathrin light chain B (CLTB), adaptor-related protein complex 2 subunit mu 1 (AP2M1), and epidermal growth factor receptor pathway substrate 15 (EPS15), in ANXA2-KO and WT PK15 cells by qRT-PCR and western-blot. RT-qPCR analysis showed that, compared with WT cells, the mRNA levels of CLTC, CLTB, AP2M1, and EPS15 were significantly upregulated in ANXA2-KO cells (Figures 6A–D), suggesting that ANXA2 deficiency disrupts the normal regulation of clathrin-mediated endocytosis-related genes. The change of mRNAs with infection time-course analysis further showed that in WT cells, EPS15 mRNA increased at 12 hpi, whereas CLTB and AP2M1 mRNA levels increased at 24 hpi, indicating JEV infection might activate the clathrin-mediated endocytic pathway during 12–24 hpi. In contrast, these changes were not observed in ANXA2-KO cells, and the mRNA expression patterns of CLTB, AP2M1, and EPS15 differed markedly from those in WT cells (Figures 6E–H). Western blot analysis of CLTC further supported these findings. In ANXA2-KO cells, CLTC protein levels were higher than those in WT cells in uninfected conditions, consistent with the RT-qPCR results (Figure 6J). In JEV infected condition, The CLTC protein gradually increased from 3 to 24 hpi in WT cells. However, CLTC expression showed an unstable pattern during infection in ANXA2-KO cells, differing substantially from that observed in WT cells (Figures 6I, J).

Because PI3K/Akt signaling is a key upstream regulator of clathrin-mediated endocytosis (Mettlen et al., 2018), we examined Akt phosphorylation during early JEV infection. In infected WT PK15 cells, Akt phosphorylation was detected as early as 1 and 3 hpi and then returned to baseline by 6 hpi, suggesting transient early activation of the PI3K/Akt pathway. In ANXA2-KO cells, Akt phosphorylation was detected at 1 hpi but was not maintained at 3 hpi, indicating impaired sustained activation of the pathway during early infection (Figures 6K, L). These results suggest that ANXA2 Knockout inhibits for continuous activation of PI3K/Akt signaling and effects the downstream regulation of clathrin-mediated endocytosis during JEV infection.

Transferrin internalization assay was performed to verify whether ANXA2 knockout impairs cellular clathrin-mediated endocytosis (CME). Wild-type and ANXA2-knockout PK15 cells were treated with 80 μM Dynasore, followed by Cy3-conjugated transferrin (Cy3-Tf) incubation and flow cytometry quantification of intracellular mean fluorescence intensity (MFI). The Cy3-Tf signal intensity was markedly reduced in ANXA2-KO cells compared with wild-type PK15 cells, whereas additional Dynasore treatment produced no further decrease of Cy3-Tf fluorescence in ANXA2-KO cells (Figure 6M). These data confirm that ANXA2 deletion significantly suppresses CME activity, which likely accounts for the reduced JEV replication observed in ANXA2-deficient cells.

### 3.7 ANXA2 knockout reduces intracellular transport of JEV during early infection

**Figure 7:**
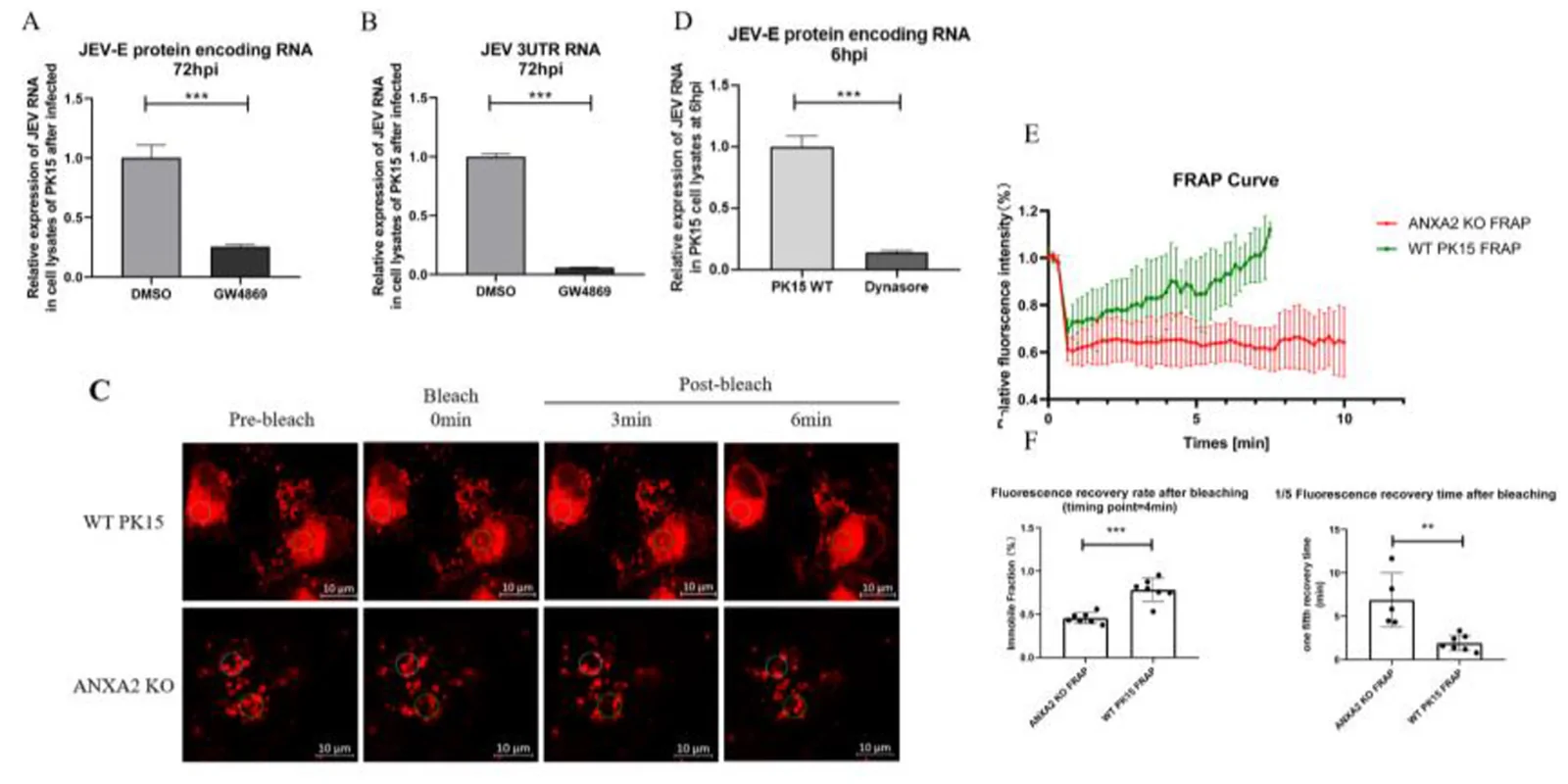
ANXA2 knockout reduces intracellular trafficking of JEV during early infection. **(A, B)** Relative levels of JEV RNA in WT and ANXA2-KO PK15 cells treated with GW4869 at 72 hpi, determined by qRT-PCR. Data are shown as mean ± SD from three independent experiments. **(C)** Representative FRAP images showing the localization of DiD-labeled JEV particles in WT and ANXA2-KO PK15 cells before and after photobleaching. The green circle indicates the bleached region, and the cyan circle indicates the reference region (non-bleached). Images were acquired every 8 s to monitor fluorescence recovery in the bleached region (*n* = 7). **(D)** The effect of 80uM Dynasore, a specific inhibitor of the clathrin-dependent endocytic pathway, on JEV infection and replication. **(E)** Relative fluorescence intensity (RFI) curves of FRAP kinetics for DiD-labeled JEV signals in WT and ANXA2-KO PK15 cells. The green line represents WT PK15 cells and the red line represents ANXA2-KO PK15 cells. Each curve represents mean value of seven biologically independent repeats. **(F)** Quantification of fluorescence recovery 4 min after photobleaching and the proportion of cells showing a fluorescence (RFI) recovery rate > 0.2. Data are shown as mean ± SD. **P < 0.01; ***P < 0.001; ns, not significant.

Based on the above findings, we hypothesized that ANXA2 may regulate JEV infection by maintaining PI3K/Akt-dependent clathrin-mediated endocytosis and intracellular transport. To examine the role of membrane trafficking in viral replication, PK15 cells were treated with GW4869, a small-molecule inhibitor of ceramide-mediated inward membrane budding and endosome/vesicle transport (Li et al., 2025), before and during JEV infection. RT-qPCR analysis showed that GW4869 treatment significantly reduced JEV 5′UTR and E gene RNA levels at 72 hpi compared with DMSO-treated controls (Figures 7A, B), indicating that intracellular membrane transport contributes to efficient JEV replication.

To investigate whether ANXA2-KO facilitates JEV replication via regulating clathrin-mediated endocytosis (CME), we treated wild-type PK15 cells with Dynasore, a specific small-molecule inhibitor of CME, and detected intracellular JEV E RNA levels at early stage of infection (6 hpi) by RT-qPCR. The results shown that treatment with 80 μM Dynasore significantly suppressed JEV RNA replication and led to markedly reduced abundance of JEV E transcripts relative to untreated control cells at the early infection stage (Figure 7D). Notably, the inhibitory effect on viral RNA abundance triggered by Dynasore was comparable to the suppression observed in ANXA2-KO cells at the same time point (Figure 5B). These data further supported that ANXA2 promotes JEV replication through functional CME pathways.

To determine whether ANXA2 affects intracellular viral transport, purified JEV particles were labeled with the lipophilic dye DiD and used to infect ANXA2-KO or WT PK15 cells. At 24 hpi, ANXA2-KO cells showed lower fluorescence intensity and fewer DiD-positive signals than WT cells, suggesting reduced uptake or retention of labeled virions (Figure 7C). These observations were consistent with the immunofluorescence and flow cytometry results described above. We used FRAP to assess intracellular mobility of labeled JEV particles in WT or KO cells at 24hpi. In WT PK15 cells, fluorescence recovered rapidly within 4–6 min after photobleaching. In contrast, fluorescence recovery was markedly delayed or absent in ANXA2-KO cells over a 10-min observation period (Figures 7C, E, F, CSV values shown as Supplementary Table 3). These FRAP results indicate that ANXA2 deficiency impairs the intracellular mobility and transport of JEV particles, which may contribute to the reduced infectivity and replication observed in ANXA2-KO cells during the early phase of infection.

## 4 Discussion

Japanese encephalitis virus (JEV) is a zoonotic pathogen with broad host tropism, capable of infecting diverse species, organs, and cell types. However, the mechanisms underlying cross-species transmission and viral infection in different host cells remain incompletely understood. As a member of the family Flaviviridae, JEV depends heavily on host cell factors to complete multiple stages of its life cycle. A variety of host proteins are involved in viral entry, RNA release and replication, virion assembly, and budding. Therefore, investigation of JEV-host protein interactions is essential not only for understanding the viral replication mechanism in host cells but also for identifying potential targets for antiviral intervention.

JEV entry is initiated by the viral envelope (E) protein, which mediates attachment to the cell surface, promotes clathrin-dependent endocytosis, and supports membrane fusion within endosomes, thereby allowing release of the viral genome into the cytoplasm (Yun and Lee, 2018). Because these processes require numerous host factors, identification of host proteins involved in the JEV life cycle is a prerequisite for understanding viral infection. Although several studies have identified attachment factors and candidate receptors in different cell types (Chen et al., 2012; Chien et al., 2008; Chiou et al., 2005; Nain et al., 2017; Niu et al., 2018; Zhao et al., 2020), relatively little is known about the cellular components that regulate JEV entry and replication. In the present study, we used IP combined with LC-MS/MS to identify annexin A2 (ANXA2) as a candidate host factor associated with JEV infection. We further examined ANXA2 expression during infection, evaluated the interaction and co-localization between ANXA2 and the JEV E protein, assessed the effect of ANXA2 blockade on viral RNA replication, and generated ANXA2 knockout (KO) PK15 cells using CRISPR/Cas9 to define the role of ANXA2 in JEV infection *in vitro*.

JEV is transmitted horizontally between arthropods and vertebrates, and pigs serve as important amplifying hosts in the natural transmission cycle. Previous studies have shown that JEV can be transmitted directly between pigs via non-vector routes, including the intranasal route, and can persist in porcine tonsils for up to 25 days, thereby facilitating viral maintenance during seasons when mosquitoes are inactive (García-Nicolás et al., 2018). These observations highlight the importance of studying JEV infection in porcine-derived cells to better understand the mechanisms of viral replication and transmission. In the present study, we used PK15 cells as a porcine host cell model to identify cellular components involved in JEV infection. Because the E protein mediates interactions with cellular attachment factors during JEV entry (Mohsin et al., 2022), we performed IP using infected PK15 cells to capture host proteins interacting with the E protein. ANXA2 was identified as a host factor associated with JEV replication by IP and LC-MS/MS. Subsequent *in vitro* validation showed that ANXA2 expression was upregulated during early stage of JEV infection, and blocking ANXA2 with increasing concentrations of antibody inhibited JEV RNA replication. In addition, ANXA2 knockout significantly impaired viral RNA accumulation at multiple time points after infection. However, confocal microscopy did not reveal widespread or obvious co-localization between the JEV E protein and ANXA2. Together, these findings suggest that ANXA2 plays an important role in JEV infection, although it may not function as a classical receptor. Rather, ANXA2 may act as an intermediate host factor that regulates cellular processes required for efficient JEV infection.

Annexins are a family of Ca^2±^ dependent phospholipid-binding proteins that bind negatively charged membrane phospholipids in a calcium-dependent manner. Their conserved core domain and variable N-terminal region enable interactions with cytoplasmic binding partners and membrane structures (White et al., 2024). Through these properties, annexins participate in endocytosis, exocytosis, and membrane remodeling by regulating membrane organization and lipid dynamics (Gerke et al., 2005). In particular, ANXA2 is localized to endosomal compartments, and specific endosome-targeting sequences have been identified within its N-terminal domain. Studies using knockdown or knockout strategies have shown that ANXA2 influences endosome morphology and endocytic transport (Wang et al., 2016). For example, ANXA2 silencing inhibits lysosomal transport of fluid-phase tracers and internalized EGF receptors (Mayran et al., 2003). Moreover, F-actin is required for transport beyond early endosomes, and the formation of actin patches on early endosomes depends on ANXA2-mediated actin nucleation and polymerization, which contributes to early-to-late endosome transport (Morel et al., 2009). These observations indicate that ANXA2 is involved not only in membrane trafficking pathways such as endocytosis and exocytosis, but also in intracellular transport between early and late endosomes. In the present study, we examined proteins involved in clathrin-mediated endocytosis in ANXA2-KO cells and found that CLTC, CLTB, AP2M1, and EPS15 were upregulated after ANXA2 knockout, suggesting that ANXA2 deficiency disrupts the balance of the clathrin-mediated endocytic pathway. In addition, inhibited activation of PI3K/Akt signaling in infected ANXA2-KO cells further supports a regulatory role for ANXA2 in early infection signaling pathways.

Accumulating evidence indicates that annexin family members can facilitate infection by diverse viruses. For example, pseudorabies virus infection promotes viral proliferation by inducing ANXA2 and Src kinase phosphorylation, thereby facilitating extracellular translocation of ANXA2; ANXA2 deficiency significantly restricts pseudorabies virus replication (Weng et al., 2023). Similarly, ANXA2 knockdown markedly reduces measles virus replication, decreases matrix protein expression, and impairs plasma membrane localization of the viral M protein (Koga et al., 2018). Hepatitis E virus ORF3 protein hijacks ANXA2 for transport to the cytoskeleton and is subsequently directed into multivesicular bodies via the nSMase-ESCRT-III pathway for secretion (Liu et al., 2025). ANXA2 has also been reported to interact with nonstructural proteins of mammalian orthoreoviruses at cellular membranes, where it contributes to maintenance of actin cytoskeleton organization and endoplasmic reticulum morphology (Tenorio et al., 2025). Notably, a previous study analyzing membrane proteins from JEV-permissive BHK-21 cells and the JEV-resistant mutant cell line 3A10-3F found that annexin 1 and annexin 2 were significantly reduced in the resistant cells (Ding et al., 2011). These findings, together with our results, support the view that ANXA2 participates in JEV attachment, entry, and intracellular trafficking.

Flaviviruses adopt divergent temporal regulatory strategies to manipulate PI3K/Akt phosphorylation for self-propagation. DENV recruits integrin-linked kinase (ILK) via viral NS1 and NS3 proteins to continuously boost Akt Ser473 phosphorylation; activated Akt subsequently triggers Erk-NF-κB signaling to elevate SOCS3 expression, which suppresses STAT1/2 phosphorylation and blocks interferon-stimulated gene transcription to evade host innate immunity (Kao et al., 2023). Previous studies have also demonstrated that the PI3K/Akt signaling pathway is involved in JEV infection, including regulation of apoptosis and inflammatory responses (Xie et al., 2021; Zhao et al., 2022). Previous research found that JEV infected upregulates PARP1 at 36 hpi, which PARylates Akt-PH domain and transactivates PTEN to suppress Akt phosphorylation, further activating FoxO1-driven autophagy to facilitate viral propagation. Consistent with previous reports, flaviviruses transiently activate Akt at early infection to restrain apoptosis; divergent late-stage Akt modulation depends on distinct host hijacking axes (Desingu et al., 2023). Our data further verify this biphasic pattern: JEV infection induces Akt phosphorylation at 1–3 hpi, while obvious suppression of Akt phosphorylation occurs from 12 to 24 hpi, perfectly matching previously reported observations. In the present study, we examined Akt phosphorylation during early JEV infection in ANXA2-KO and WT PK15 cells. In WT cells, Akt phosphorylation was detected during the early phase of infection (1, 3 hpi), suggesting rapid activation of the PI3K/Akt pathway shortly after JEV entry. In contrast, ANXA2-KO cells showed impaired sustained Akt phosphorylation activation of the pathway during early infection (only phosphorylation at 1 hpi), indicating dysregulated PI3K/Akt signaling in the absence of ANXA2. Many viruses are known to manipulate the PI3K/Akt cascade indirectly by altering host protein expression. Our findings suggest that ANXA2 knockout disrupts JEV-induced regulation of PI3K/Akt signaling, which may contribute to the abnormal expression of downstream clathrin-mediated endocytosis-related proteins.

Fluorescence recovery after photobleaching (FRAP) is a useful technique for assessing intracellular membrane fluidity and molecular transport and has been widely applied to study viral material transport in infected cells. For example, fluorescent *in situ* hybridization combined with FRAP has been used to analyze the mobility and diffusion of influenza A virus mRNA in live cells (Wang et al., 2008). In this study, we used DiD, a long-chain lipophilic carbocyanine dye, to label concentrated JEV particles before infection of PK15 cells. DiD readily inserts into lipid membranes, including cellular membranes and viral envelopes, and can therefore be used to monitor virus binding, membrane fusion, and intracellular trafficking (Ayala-Nuñez et al., 2011). After infection with DiD-labeled JEV, ANXA2-KO and WT PK15 cells were examined by live-cell imaging and FRAP at 24 hpi. We found that DiD fluorescence intensity was lower in ANXA2-KO cells, and fluorescence recovery after bleaching was significantly delayed compared with WT cells, indicating reduced intracellular transport capacity. Although virus concentration from cell supernatants may also include exosome-like particles that are difficult to fully remove by ultracentrifugation, this does not alter the interpretation that ANXA2 deficiency impairs the uptake and trafficking of lipid-associated particles, including virions and potentially exosome-like vesicles. Thus, ANXA2 appears to contribute to the intracellular mobility of JEV-containing particles.

Regretfully, all experiments in this study adopted the attenuated JEV vaccine strain SA14-14-2 rather than virulent wild-type strains, which constitutes a major limitation. Although its E protein sequence is highly conserved with virulent isolates and the attenuation mutations do not disrupt ANXA2-E binding domains, viral replication and host factor dependence may differ between vaccine and neurovirulent strains. Our conclusions require further verification with virulent JEV strains in primary porcine cells or animal models. Future work will explore ANXA2 regulatory roles during infection by wild-type isolates virulent JEV to extend the translational value of these findings.

In summary, we used IP and LC-MS/MS to identify host proteins interacting with the JEV E protein in permissive PK15 cells and identified ANXA2 as a host factor required for efficient JEV replication. Using CRISPR/Cas9-generated ANXA2-KO PK15 cells, we demonstrated that ANXA2 deficiency significantly reduced viral RNA replication, especially during the early stage of JEV infection (2-6hpi), when the viral entry and initiates replication. Moreover, ANXA2 knockout disrupted the expression of clathrin-mediated endocytosis-related proteins, altered Akt phosphorylation during early infection, and reduced the internalization and intracellular transport efficiency of JEV in PK15 cells. Collectively, these findings indicate that ANXA2 plays an important role in JEV infection and endocytic trafficking. Further studies are needed to define the precise molecular mechanism by which JEV exploits ANXA2, but ANXA2 may represent a promising target for antiviral intervention against JEV.

## Data Availability

The mass spectrometry proteomics raw data and quantitative tables generated in this study have been deposited in the PRIDE repository via the ProteomeXchange Consortium under dataset identifier PXD081082 (https://www.ebi.ac.uk/pride/archive/projects/PXD081082). The other original data presented in the study are included in the article/Supplementary material, further inquiries can be directed to the corresponding author.

## Supplementary material

Supplementary Figure S1 **(A–C)**: Confocal immunofluorescence analysis of co-localization between JEV E protein and ANXA2 in infected PK15 cells at 12hpi. JEV E protein was detected with iFluor 488-conjugated goat anti-rabbit IgG (green), ANXA2 was detected with iFluor 594-conjugated goat anti-rabbit IgG (red), and nuclei were counterstained with DAPI (blue). Pearson's correlation coefficient was calculated using Fiji/ImageJ. Scale bar, 10 μm. **(D)** Co-immunoprecipitation of ANXA2 and JEV E protein using anti-ANXA2 polyclonal antibody as the IP antibody and anti-JEV E monoclonal antibody to western-blot detected. IP antibody incubation time to 6 h, the anti-tubulin as the reference control. **(E)** SDS-PAGE followed by Coomassie Brilliant Blue staining of samples collected during immunoprecipitation, including wash buffer, flow-through buffer, and elution buffer.
