# Supplementary material for: Annexin A2 facilitates Japanese encephalitis virus replication by regulating endocytosis and intracellular transport in PK15 cells
Source: Front Microbiol. 2026 Aug 7;17:1858383. doi: 10.3389/fmicb.2026.1858383 (PMC13493599; doi:10.3389/fmicb.2026.1858383)
Supplement: Supplementary file 1 [file Data_Sheet_1.PDF]

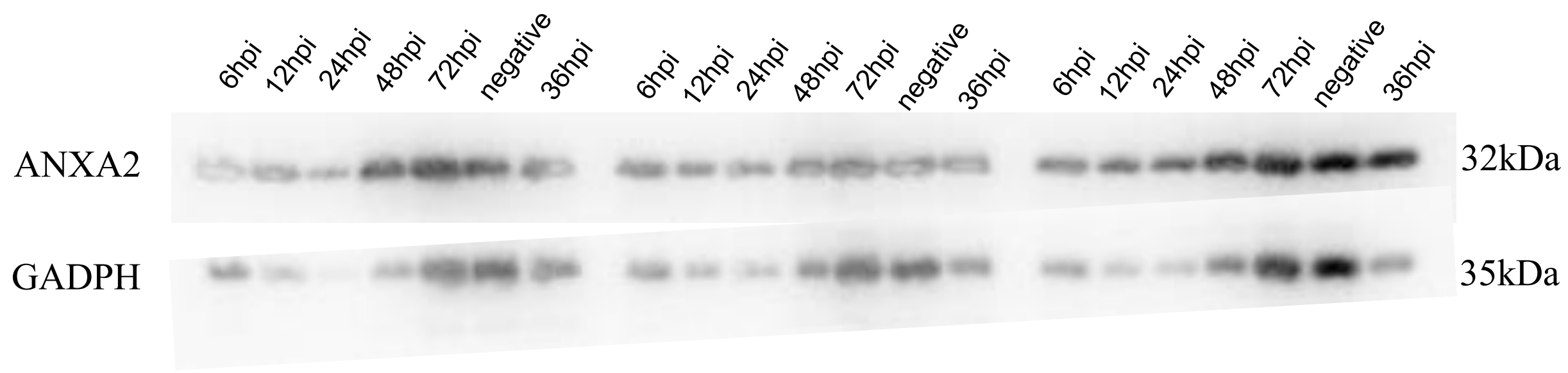
6hpi
12hpi
24hpi
48hpi
72hpi
negative
36hpi
6hpi
12hpi
24hpi
48hpi
72hpi
negative
36hpi
6hpi
12hpi
24hpi
48hpi
72hpi
negative
36hpi
ANXA2
32kDa
GADPH
35kDa

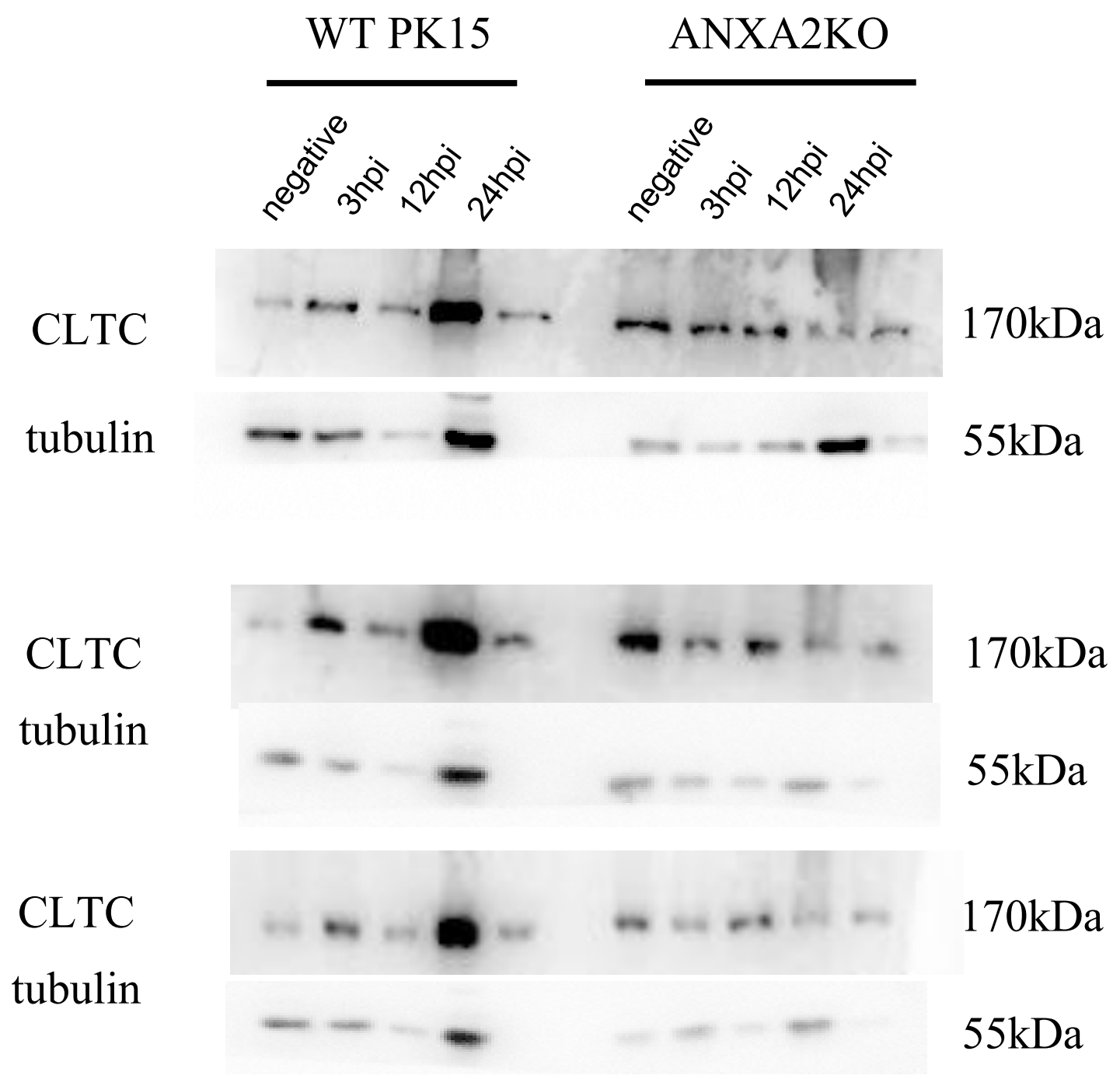
WT PK15
ANXA2KO
negative
3hpi
12hpi
24hpi
negative
3hpi
12hpi
24hpi
CLTC
170kDa
tubulin
55kDa
CLTC
170kDa
tubulin
55kDa
CLTC
170kDa
tubulin
55kDa

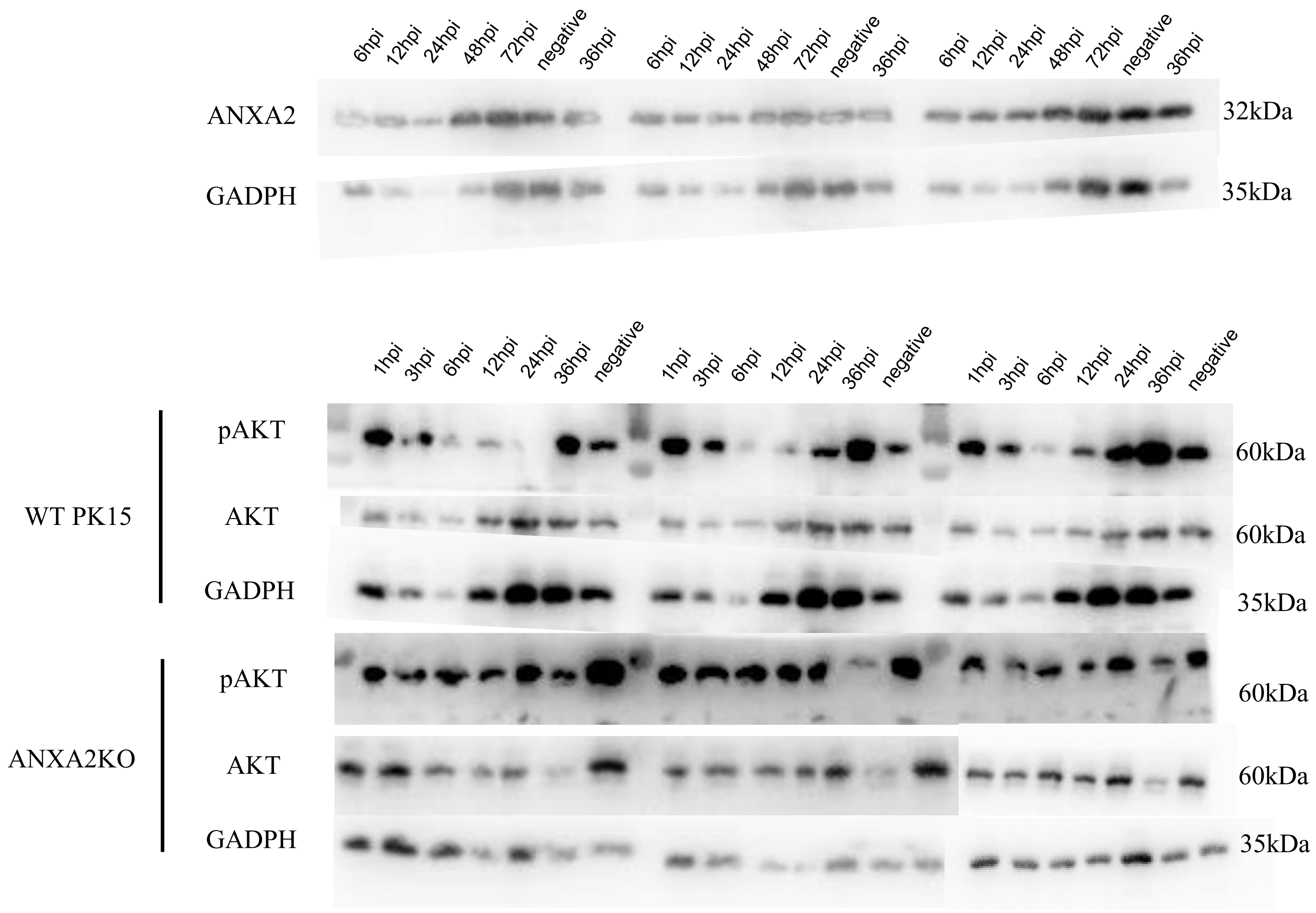
6hpi
12hpi
24hpi
48hpi
72hpi
negative
36hpi
6hpi
12hpi
24hpi
48hpi
72hpi
negative
36hpi
6hpi
12hpi
24hpi
48hpi
72hpi
negative
36hpi
ANXA2
32kDa
GADPH
35kDa
1hpi
3hpi
6hpi
12hpi
24hpi
36hpi
negative
1hpi
3hpi
6hpi
12hpi
24hpi
36hpi
negative
1hpi
3hpi
6hpi
12hpi
24hpi
36hpi
negative
WT PK15
pAKT
60kDa
AKT
60kDa
GADPH
35kDa
ANXA2KO
pAKT
60kDa
AKT
60kDa
GADPH
35kDa
