# Supplementary material for: Annexin A2 facilitates Japanese encephalitis virus replication by regulating endocytosis and intracellular transport in PK15 cells
Source: Front Microbiol. 2026 Aug 7;17:1858383. doi: 10.3389/fmicb.2026.1858383 (PMC13493599; doi:10.3389/fmicb.2026.1858383)
Supplement: Supplementary file 2 [file Table_1.DOCX]

Supplementary Table 1

Primer sequences for RT-qPCR analyses

| Gene | Primers sequence (5‘-3’) | Gene accession | Length (bp) |
| --- | --- | --- | --- |
| β-actin | F: CTCCATCATGAAGTGCGACGT | NM_001444420.1 | 114 |
|  | R: GTGATCTCCTTCTGCATCCTGTC |  |  |
| JEV E | F: CGGTGGACACTGGTCACGG | KX254417.1 | 160 |
|  | R: CTCGACCAGCACCTTTGAGTT |  |  |
| JEV 3UTR | F: CCAGTCTATTCCCAGGTGTCAA | KX254417.1 | 84 |
|  | R: GCTGTAGAGGAGGTGGAAGGAC |  |  |
| CLTC | F: GATCTCTTTGAATACCGTTGC | NM_001146127.1 | 168 |
|  | R: GCCAGTCAGAAGTAACCAC |  |  |
| AP2M1 | F: GCCGATGAAACAAGCAAGAG | NM_001243644.1 | 102 |
|  | R: GATGCTGCGTTCAGAGTCAA |  |  |
| ESP15 | F: ATTCTTTGTTGCTTTGCGTCTTG | XM_021096669.1 | 186 |
|  | R: GCATCATATTTGGCCTTATCTTC |  |  |
| CLTB | F: AGCTGGATGCTGCCTCCAAAGTG | XM_021084433.1 | 79 |
|  | R: CGCTGGTTCCATTCCTCCAAGTC |  |  |
|  |  |  |  |
|  |  |  |  |
|  |  |  |  |
