# Supplementary material for: Annexin A2 facilitates Japanese encephalitis virus replication by regulating endocytosis and intracellular transport in PK15 cells
Source: Front Microbiol. 2026 Aug 7;17:1858383. doi: 10.3389/fmicb.2026.1858383 (PMC13493599; doi:10.3389/fmicb.2026.1858383)
Supplement: Supplementary file 3 [file Table_2.DOCX]

Supplementary Table 2

Designed gRNA for KnockOut ANXA2 by CRISPR/Cas9

| Oligo name | Forward primers | Reverse primers |
| --- | --- | --- |
| gRNA1 | GCGCTGAAGTCAGCCTTGTC | GACAAGGCTGACTTCAGCGC |
| gRNA2 | CCAAAATCACTGTCTCCAGG | CCTGGAGACAGTGATTTTGG |
| ANXA2KO | F: CCAGTCTATTCCCAGGTGTCAA | GCTGTAGAGGAGGTGGAAGGAC |
|  |  |  |
|  |  |  |
