# Supplementary material for: Annexin A2 facilitates Japanese encephalitis virus replication by regulating endocytosis and intracellular transport in PK15 cells
Source: Front Microbiol. 2026 Aug 7;17:1858383. doi: 10.3389/fmicb.2026.1858383 (PMC13493599; doi:10.3389/fmicb.2026.1858383)
Supplement: Supplementary file 4 [file Table_3.DOCX]

Supplementary Table 3

FRAP images CSV values in different times

| ANXA2 KO FRAP Regions CSV values | | | | | | | | | | | | | | |
| --- | --- | --- | --- | --- | --- | --- | --- | --- | --- | --- | --- | --- | --- | --- |
| samples name | | A2KO 3h DiD FRAP Image21 | | | A2KO 3h DiD FRAP Image28 | | | A2KO 3h DiD FRAP Image26 | | | A2KO 3h DiD FRAP Image36 | | |  |
| Time [s] | Time [min] | Bleach Region 1 | control Region 2 | blank Region 3 | Bleach Region 1 | control Region 2 | blank Region 3 | Bleach Region 1 | control Region 2 | blank Region 3 | Bleach Region 1 | control Region 2 | blank Region 3 |  |
| 0 | 0 | 68.374 | 54.152 | 0.538 | 107.605 | 62.743 | 0.211 | 170.677 | 187.762 | 0.216 | 188.597 | 143.4 | 0.184 |  |
| 9.956 | 0.165933 | 67.399 | 54.912 | 0.5 | 105.293 | 60.558 | 0.37 | 174.929 | 188.807 | 0.173 | 189.715 | 145.292 | 0.175 |  |
| 19.956 | 0.3326 | 66.594 | 59.551 | 0.505 | 100.984 | 61.927 | 0.295 | 170.507 | 186.824 | 0.187 | 190.983 | 144.98 | 0.193 |  |
|  |  | bleaching |  |  | bleaching |  |  | bleaching |  |  | bleaching |  |  |  |
| 39.268 | 0.654467 | 34.222 | 65.967 | 0.519 | 64.136 | 61.563 | 0.253 |  |  |  | 103.71 | 150.803 | 0.182 |  |
| 49.213 | 0.820217 | 31.601 | 69.634 | 0.499 | 60.338 | 61.654 | 0.299 | 80.528 | 187.991 | 0.193 | 101.766 | 149.912 | 0.186 |  |
| 59.167 | 0.986117 | 34.874 | 70.634 | 0.565 | 61.181 | 61.441 | 0.268 | 80.191 | 186.623 | 0.172 | 101.3 | 148.082 | 0.204 |  |
| 69.167 | 1.152783 | 36.76 | 67.397 | 0.448 | 60.25 | 63.577 | 0.212 | 81.101 | 187.122 | 0.218 | 103.584 | 153.796 | 0.208 |  |
| 79.168 | 1.319467 | 38.326 | 65.861 | 0.561 | 62.714 | 62.291 | 0.345 | 84.648 | 186.449 | 0.2 | 107.932 | 153.849 | 0.202 |  |
| 89.167 | 1.486117 | 40.305 | 64.258 | 0.549 | 62.029 | 62.375 | 0.275 | 83.376 | 187.261 | 0.232 | 107.779 | 152.655 | 0.203 |  |
| 99.167 | 1.652783 | 44.242 | 63.684 | 0.476 | 63.846 | 62.327 | 0.332 | 84.251 | 185.646 | 0.207 | 107.612 | 150.82 | 0.188 |  |
| 109.166 | 1.819433 | 44.97 | 63.04 | 0.466 | 64.625 | 61.143 | 0.261 | 83.32 | 181.83 | 0.187 | 104.749 | 150.192 | 0.228 |  |
| 119.166 | 1.9861 | 45.634 | 66.273 | 0.496 | 66.537 | 61.242 | 0.347 | 82.498 | 179.264 | 0.179 | 102.394 | 150.736 | 0.248 |  |
| 129.168 | 2.1528 | 44.738 | 63.601 | 0.414 | 65.63 | 61.455 | 0.307 | 83.166 | 177.304 | 0.234 | 102.719 | 147.702 | 0.218 |  |
| 139.168 | 2.319467 | 46.547 | 66.223 | 0.492 | 69.155 | 60.575 | 0.305 | 80.691 | 175.344 | 0.164 | 101.988 | 143.292 | 0.221 |  |
| 149.167 | 2.486117 | 43.356 | 69.375 | 0.444 | 69.568 | 59.924 | 0.272 | 83.004 | 175.405 | 0.21 | 102.024 | 142.873 | 0.235 |  |
| 159.166 | 2.652767 | 42.981 | 68.173 | 0.513 | 70.809 | 58.729 | 0.374 | 83.42 | 172.502 | 0.192 | 105.177 | 145.084 | 0.166 |  |
| 169.169 | 2.819483 | 40.634 | 65.349 | 0.537 | 69.212 | 59.566 | 0.306 | 81.181 | 178.163 | 0.21 | 108.962 | 143.236 | 0.22 |  |
| 179.168 | 2.986133 | 40.903 | 62.554 | 0.506 | 67.612 | 58.167 | 0.194 | 79.731 | 175.193 | 0.171 | 102.874 | 143.075 | 0.193 |  |
| 189.167 | 3.152783 | 40.822 | 64.563 | 0.406 | 69.738 | 60.199 | 0.343 | 80.037 | 173.496 | 0.153 | 103.721 | 141.034 | 0.178 |  |
| 199.167 | 3.31945 | 41.693 | 64.497 | 0.497 | 74.123 | 59.598 | 0.262 | 81.136 | 174.6 | 0.209 | 104.397 | 147.687 | 0.174 |  |
| 209.168 | 3.486133 | 42.051 | 62.942 | 0.379 | 72.805 | 57.567 | 0.308 | 82.604 | 169.689 | 0.172 | 103.181 | 153.231 | 0.174 |  |
| 219.167 | 3.652783 | 41.907 | 65.002 | 0.417 | 74.91 | 61.028 | 0.296 | 79.809 | 172.678 | 0.165 | 103.373 | 142.565 | 0.228 |  |
| 229.167 | 3.81945 | 42.013 | 59.067 | 0.425 | 72.513 | 60.86 | 0.304 | 78.303 | 171.723 | 0.207 | 100.806 | 140.428 | 0.2 |  |
| 239.167 | 3.986117 | 41.604 | 58.117 | 0.48 | 73.462 | 59.063 | 0.369 | 79.83 | 169.247 | 0.258 | 101.011 | 139.868 | 0.175 |  |
| 249.166 | 4.152767 | 42.217 | 57.455 | 0.449 | 75.317 | 58.551 | 0.272 | 80.105 | 167.567 | 0.174 | 100.744 | 140.234 | 0.308 |  |
| 259.167 | 4.31945 | 43.77 | 59.187 | 0.413 | 74.872 | 59.122 | 0.268 | 78.065 | 171.616 | 0.21 | 106.558 | 144.297 | 0.169 |  |
| 269.168 | 4.486133 | 43.095 | 58.804 | 0.424 | 75.832 | 56.592 | 0.263 | 76.836 | 170.88 | 0.188 | 105.204 | 141.882 | 0.132 |  |
| 279.168 | 4.6528 | 42.466 | 62.071 | 0.475 | 77.124 | 59.21 | 0.224 | 75.144 | 172.266 | 0.177 | 107.903 | 143.677 | 0.226 |  |
| 289.167 | 4.81945 | 42.805 | 60.488 | 0.413 | 76.263 | 61.904 | 0.287 | 74.883 | 165.203 | 0.174 | 107.381 | 145.839 | 0.177 |  |
| 299.167 | 4.986117 | 41.668 | 64.393 | 0.425 | 76.107 | 59.625 | 0.3 | 75.91 | 166.189 | 0.161 | 112.784 | 146.128 | 0.204 |  |
| 309.168 | 5.1528 | 39.904 | 67.109 | 0.392 | 71.444 | 59.59 | 0.227 | 74.327 | 166.319 | 0.18 | 112.011 | 144.878 | 0.239 |  |
| 319.167 | 5.31945 | 41.788 | 68 | 0.459 | 68.765 | 60.28 | 0.26 | 75.054 | 166.057 | 0.23 | 113.78 | 144.634 | 0.169 |  |
| 329.166 | 5.4861 | 38.917 | 63.465 | 0.468 | 66.545 | 57.324 | 0.277 | 77.1 | 165.098 | 0.193 | 115.261 | 142.177 | 0.204 |  |
| 339.167 | 5.652783 | 39.011 | 61.744 | 0.426 | 70.081 | 57.008 | 0.286 | 75.735 | 161.44 | 0.237 | 111.167 | 142.006 | 0.177 |  |
| 349.168 | 5.819467 | 39.189 | 65.32 | 0.498 | 68.683 | 54.63 | 0.361 | 75.087 | 160.857 | 0.172 | 114.392 | 140.076 | 0.159 |  |
| 359.167 | 5.986117 | 39.013 | 61.187 | 0.415 | 63.825 | 56.558 | 0.309 | 74.616 | 161.196 | 0.166 | 116.084 | 143.296 | 0.246 |  |
| 369.167 | 6.152783 | 41.744 | 62.166 | 0.542 | 64.665 | 55.956 | 0.344 | 72.141 | 161.456 | 0.17 | 109.902 | 138.388 | 0.152 |  |
| 379.166 | 6.319433 | 39.834 | 65.234 | 0.53 | 64.036 | 56.406 | 0.243 | 71.16 | 155.864 | 0.203 | 113.363 | 140.18 | 0.159 |  |
| 389.168 | 6.486133 | 38.521 | 71.185 | 0.426 | 63.144 | 54.666 | 0.312 | 72.156 | 153.981 | 0.159 | 111.679 | 137.32 | 0.21 |  |
| 399.167 | 6.652783 | 36.952 | 70.44 | 0.46 | 65.835 | 56.403 | 0.291 | 72.486 | 153.907 | 0.183 | 114.834 | 142.597 | 0.238 |  |
| 409.168 | 6.819467 | 36.253 | 69.735 | 0.487 | 65.672 | 56.115 | 0.273 | 72.406 | 155.897 | 0.202 | 113.898 | 142.279 | 0.277 |  |
| 419.167 | 6.986117 | 36.272 | 72.615 | 0.379 | 64.913 | 57.49 | 0.251 | 73.748 | 156.862 | 0.197 | 109.199 | 146.01 | 0.223 |  |
| 429.167 | 7.152783 | 35.481 | 72.665 | 0.429 | 65.296 | 56.466 | 0.305 | 76.806 | 159.788 | 0.213 | 107.873 | 144.927 | 0.165 |  |
| 439.166 | 7.319433 | 35.227 | 68.166 | 0.526 | 66.765 | 59.247 | 0.278 | 77.039 | 159.005 | 0.166 | 107.737 | 145.214 | 0.192 |  |
| 449.168 | 7.486133 | 37.01 | 70.584 | 0.39 | 67.754 | 60.6 | 0.319 | 76.853 | 157.884 | 0.186 | 106.517 | 148.308 | 0.198 |  |
| 459.167 | 7.652783 | 35.542 | 66.518 | 0.547 | 69.005 | 60.519 | 0.321 | 76.512 | 159.244 | 0.214 | 103.504 | 146.349 | 0.213 |  |
| 469.167 | 7.81945 | 35.724 | 61.47 | 0.475 | 71.914 | 54.514 | 0.299 | 77.763 | 161.807 | 0.151 | 104.856 | 143.571 | 0.195 |  |
| 479.168 | 7.986133 | 37.586 | 60.708 | 0.542 | 70.295 | 53.974 | 0.248 | 79.011 | 161.842 | 0.197 | 101.714 | 141.307 | 0.235 |  |
| 489.167 | 8.152783 | 37.533 | 61.341 | 0.474 | 65.835 | 49.961 | 0.289 | 81.56 | 161.484 | 0.172 | 101.102 | 140.116 | 0.341 |  |
| 499.167 | 8.31945 | 38.183 | 55.627 | 0.496 | 64.424 | 50.209 | 0.26 | 77.708 | 159.123 | 0.205 | 97.425 | 138.542 | 0.16 |  |
| 509.167 | 8.486117 | 34.963 | 53.521 | 0.392 | 64.683 | 48.064 | 0.3 | 78.808 | 154.136 | 0.139 | 96.773 | 138.873 | 0.203 |  |
| 519.168 | 8.6528 | 35.663 | 55.933 | 0.529 | 63.243 | 48.814 | 0.23 | 78.531 | 154.918 | 0.168 | 94.575 | 135.594 | 0.214 |  |
| 529.167 | 8.81945 | 34.891 | 58.224 | 0.491 | 63.007 | 48.562 | 0.196 | 75.824 | 152.586 | 0.175 | 92.812 | 136.184 | 0.208 |  |
| 539.167 | 8.986117 | 33.703 | 55.601 | 0.47 | 61.632 | 50.281 | 0.238 | 74.266 | 150.278 | 0.152 | 90.739 | 135.052 | 0.233 |  |
| 549.167 | 9.152783 | 33.125 | 55.915 | 0.527 | 62.768 | 47.529 | 0.289 | 74.426 | 151.562 | 0.158 | 93.089 | 127.314 | 0.242 |  |
| 559.168 | 9.319467 | 32.723 | 59.785 | 0.503 | 60.738 | 46.489 | 0.276 | 76.427 | 150.733 | 0.21 | 93.389 | 126.141 | 0.224 |  |
| 569.167 | 9.486117 | 37.037 | 56.554 | 0.486 | 60.185 | 46.865 | 0.259 | 76.51 | 150.168 | 0.208 | 93.346 | 121.803 | 0.212 |  |
| 579.168 | 9.6528 | 35.303 | 55.53 | 0.391 |  |  |  | 77.146 | 150.809 | 0.143 | 91.126 | 125.723 | 0.187 |  |
| 589.167 | 9.81945 | 36.372 | 56.149 | 0.405 |  |  |  | 78.327 | 148.295 | 0.2 | 87.721 | 122.919 | 0.163 |  |
| 599.167 | 9.986117 | 34.833 | 57.38 | 0.414 |  |  |  | 81.72 | 144.827 | 0.162 | 90.036 | 124.337 | 0.221 |  |
| 609.167 | 10.15278 |  |  |  |  |  |  | 79.327 | 140.108 | 0.197 | 88.796 | 122.61 | 0.163 |  |
| 619.168 | 10.31947 |  |  |  |  |  |  | 82.768 | 141.417 | 0.215 | 88.563 | 122.781 | 0.175 |  |
| 629.167 | 10.48612 |  |  |  |  |  |  | 80.651 | 138.603 | 0.183 | 87.786 | 121.209 | 0.166 |  |
| 639.167 | 10.65278 |  |  |  |  |  |  | 76.847 | 138.075 | 0.203 | 87.335 | 120.496 | 0.251 |  |
| 649.168 | 10.81947 |  |  |  |  |  |  | 77.307 | 134.098 | 0.221 | 83.632 | 120.615 | 0.195 |  |
| 659.167 | 10.98612 |  |  |  |  |  |  | 76.452 | 133.645 | 0.207 | 85.876 | 119.866 | 0.158 |  |
| 669.167 | 11.15278 |  |  |  |  |  |  | 74.9 | 131.456 | 0.171 | 86.097 | 119.691 | 0.221 |  |
| 679.168 | 11.31947 |  |  |  |  |  |  | 77.594 | 132.214 | 0.225 | 85.065 | 117.609 | 0.211 |  |
| 689.168 | 11.48613 |  |  |  |  |  |  | 77.709 | 130.8 | 0.185 | 83.24 | 118.403 | 0.251 |  |
| 699.167 | 11.65278 |  |  |  |  |  |  | 76.149 | 130.163 | 0.197 | 84.308 | 121.507 | 0.398 |  |

| PK15 FRAP Regions CSV values | | | | | | | | | | | | | |
| --- | --- | --- | --- | --- | --- | --- | --- | --- | --- | --- | --- | --- | --- |
| samples name | | PK15 3h DiD FRAP Image10 | | | PK15 3h DiD FRAP Image15 | | | PK15 3h DiD FRAP Image23 | | | PK15 3h DiD FRAP Image9 | | |
| Time [s] | Time [min] | Bleach Region 1 | control Region 2 | blank Region 3 | Bleach Region 1 | control Region 2 | blank Region 3 | Bleach Region 1 | control Region 2 | blank Region 3 | Bleach Region 1 | control Region 2 | blank Region 3 |
| 0 | 0 | 226.492 | 168.087 | 0.324 | 217.025 | 226.647 | 0.115 | 108.734 | 113.065 | 0.138 | 68.784 | 57.798 | 64.98 |
| 9.956 | 0.165933 | 230.437 | 169.229 | 0.347 | 215.086 | 226.443 | 0.148 | 112.187 | 114.865 | 0.136 | 65.738 | 60.063 | 64.537 |
| 19.956 | 0.3326 | 235.464 | 170.003 | 0.386 | 212.541 | 224.456 | 0.115 | 113.56 | 116.211 | 0.162 | 66.929 | 59.899 | 66.029 |
|  |  | bleaching |  |  | bleaching |  |  | bleaching |  |  | bleaching |  |  |
| 39.268 | 0.654467 | 177.025 | 171.349 | 0.262 | 143.706 | 214.088 | 0.093 | 80.086 | 119.872 | 0.12 | 43.923 | 59.785 | 66.773 |
| 49.213 | 0.820217 | 186.934 | 165.889 | 0.236 | 147.75 | 209.153 | 0.095 | 82.163 | 118.865 | 0.135 | 45.003 | 60.409 | 66.051 |
| 59.167 | 0.986117 | 193.028 | 165.8 | 0.167 | 144.298 | 205.463 | 0.131 | 82.065 | 119.229 | 0.139 | 44.384 | 60.848 | 66.888 |
| 69.167 | 1.152783 | 204.919 | 164.964 | 0.174 | 141.051 | 202.158 | 0.124 | 82.752 | 118.739 | 0.153 | 42.912 | 58.756 | 68.022 |
| 79.168 | 1.319467 | 208.101 | 163.632 | 0.197 | 141.474 | 200.109 | 0.116 | 84.173 | 117.246 | 0.129 | 44.018 | 58.764 | 71.18 |
| 89.167 | 1.486117 | 209.814 | 161.394 | 0.176 | 138.852 | 198.209 | 0.114 | 84.915 | 119.113 | 0.122 | 43.87 | 58.07 | 69.444 |
| 99.167 | 1.652783 | 214.403 | 162.759 | 0.139 | 134.617 | 196.149 | 0.097 | 85.196 | 120.616 | 0.142 | 44.25 | 56.707 | 69.165 |
| 109.166 | 1.819433 | 219.152 | 165.929 | 0.123 | 139.934 | 197.075 | 0.069 | 84.873 | 122.543 | 0.139 | 44.961 | 57.084 | 68.435 |
| 119.166 | 1.9861 | 220.15 | 165.054 | 0.147 | 139.892 | 195.181 | 0.14 | 88.396 | 126.28 | 0.152 | 45.997 | 58.113 | 69.277 |
| 129.168 | 2.1528 | 221.575 | 164.943 | 0.212 | 140.263 | 196.924 | 0.119 | 91.48 | 126.772 | 0.141 | 47.278 | 58.949 | 68.919 |
| 139.168 | 2.319467 | 223.31 | 166.764 | 0.083 | 144.321 | 200.011 | 0.279 | 90.954 | 128.773 | 0.143 | 48.842 | 57.421 | 67.899 |
| 149.167 | 2.486117 | 223.336 | 166.661 | 0.179 | 147.474 | 201.778 | 0.323 | 92.979 | 133.662 | 0.131 | 50.387 | 55.387 | 68.304 |
| 159.166 | 2.652767 | 222.301 | 167.512 | 0.189 | 148.367 | 204.828 | 0.268 | 94.135 | 136.626 | 0.141 | 49.201 | 54.68 | 66.179 |
| 169.169 | 2.819483 | 224.434 | 164.297 | 0.21 | 154.034 | 208.454 | 0.258 | 95.632 | 137.464 | 0.16 | 50.328 | 54.63 | 67.439 |
| 179.168 | 2.986133 | 220.917 | 164.767 | 0.226 | 163.442 | 216.144 | 0.316 | 99.18 | 140.058 | 0.121 | 55.252 | 55.344 | 67.344 |
| 189.167 | 3.152783 | 220.017 | 159.91 | 0.158 | 167.609 | 218.666 | 0.172 | 103.719 | 145.044 | 0.115 | 54.91 | 56.094 | 67.328 |
| 199.167 | 3.31945 | 215.618 | 152.603 | 0.143 | 168.69 | 219.71 | 0.065 | 107.261 | 147.107 | 0.13 | 54.311 | 55.034 | 68.392 |
| 209.168 | 3.486133 | 210.257 | 148.27 | 0.154 | 173.407 | 223.003 | 0.241 | 109.92 | 149.415 | 0.143 | 53.189 | 55.653 | 68.704 |
| 219.167 | 3.652783 | 209.988 | 145.738 | 0.237 | 179.61 | 227.011 | 0.212 | 110.704 | 150.297 | 0.122 | 51.881 | 54.003 | 67.834 |
| 229.167 | 3.81945 | 194.488 | 128.782 | 0.133 | 183.196 | 229.292 | 0.38 | 112.183 | 149.683 | 0.134 | 52.871 | 52.06 | 68.989 |
| 239.167 | 3.986117 | 187.418 | 122.771 | 0.152 | 185.446 | 229.984 | 0.363 | 114.049 | 151.165 | 0.122 | 50.011 | 51.863 | 67.619 |
| 249.166 | 4.152767 | 179.832 | 115.825 | 0.108 | 188.822 | 232.624 | 0.692 | 116.797 | 150.337 | 0.121 | 48.858 | 48.871 | 66.296 |
| 259.167 | 4.31945 |  |  |  | 198.527 | 234.917 | 0.796 | 115.859 | 149.079 | 0.098 | 47.531 | 47.807 | 63.545 |
| 269.168 | 4.486133 |  |  |  | 197.9 | 235.779 | 0.863 | 116.915 | 149.774 | 0.119 | 47.47 | 46.84 | 63.45 |
| 279.168 | 4.6528 |  |  |  | 198.92 | 236.42 | 0.816 | 118.062 | 149.381 | 0.109 | 48.144 | 46.014 | 63.209 |
| 289.167 | 4.81945 |  |  |  | 202.116 | 236.915 | 0.933 | 119.652 | 148.223 | 0.111 | 45.869 | 47.66 | 63.616 |
| 299.167 | 4.986117 |  |  |  | 203.022 | 239.249 | 1.041 | 119.924 | 146.688 | 0.094 | 46.974 | 45.232 | 62.978 |
| 309.168 | 5.1528 |  |  |  | 204.012 | 239.865 | 1.009 | 117.888 | 145.64 | 0.101 | 47.764 | 43.618 | 62.909 |
| 319.167 | 5.31945 |  |  |  | 207.722 | 239.897 | 1.225 | 117.867 | 145.005 | 0.112 | 46.278 | 43.285 | 62.319 |
| 329.166 | 5.4861 |  |  |  | 211.235 | 242.308 | 1.11 | 116.86 | 143.267 | 0.097 | 46.653 | 41.304 | 60.095 |
| 339.167 | 5.652783 |  |  |  | 213.531 | 242.044 | 1.225 | 116.536 | 141.941 | 0.106 | 47.107 | 41.726 | 59.783 |
| 349.168 | 5.819467 |  |  |  | 214.838 | 242.935 | 1.248 | 114.881 | 140.589 | 0.085 | 46.382 | 42.573 | 59.287 |
| 359.167 | 5.986117 |  |  |  | 215.147 | 242.201 | 1.065 | 115.332 | 138.706 | 0.103 | 47.081 | 41.066 | 59.623 |
| 369.167 | 6.152783 |  |  |  | 215.474 | 243.065 | 1.05 | 114.988 | 140.796 | 0.084 | 45.619 | 40.917 | 56.852 |
| 379.166 | 6.319433 |  |  |  | 214.559 | 241.723 | 0.945 | 114.585 | 141.682 | 0.091 | 47.749 | 40.096 | 55.805 |
| 389.168 | 6.486133 |  |  |  |  |  |  | 115.104 | 139.984 | 0.074 | 46.911 | 41.046 | 56.55 |
| 399.167 | 6.652783 |  |  |  |  |  |  | 114.892 | 136.867 | 0.062 | 47.484 | 41.757 | 57.847 |
| 409.168 | 6.819467 |  |  |  |  |  |  | 114.006 | 138.564 | 0.105 | 48.685 | 40.31 | 58.872 |
| 419.167 | 6.986117 |  |  |  |  |  |  | 112.185 | 137.328 | 0.101 | 47.397 | 39.868 | 59.251 |
| 429.167 | 7.152783 |  |  |  |  |  |  | 112.096 | 136.335 | 0.083 | 47.721 | 39.78 | 58.115 |
| 439.166 | 7.319433 |  |  |  |  |  |  | 110.332 | 134.572 | 0.097 | 49.681 | 40.122 | 57.831 |
| 449.168 | 7.486133 |  |  |  |  |  |  |  |  |  | 52.808 | 40.872 | 57.556 |
| 459.167 | 7.652783 |  |  |  |  |  |  |  |  |  | 51.314 | 40.955 | 58.254 |
| 469.167 | 7.81945 |  |  |  |  |  |  |  |  |  | 50.548 | 42.024 | 58.524 |
| 479.168 | 7.986133 |  |  |  |  |  |  |  |  |  | 49.031 | 40.988 | 57.558 |
